# Molecular imaging of bacterial outer membrane vesicles based on bacterial surface display

**DOI:** 10.1038/s41598-023-45628-9

**Published:** 2023-10-31

**Authors:** Dávid Szöllősi, Polett Hajdrik, Hedvig Tordai, Ildikó Horváth, Dániel S. Veres, Bernadett Gillich, Kanni Das Shailaja, László Smeller, Ralf Bergmann, Michael Bachmann, Judith Mihály, Anikó Gaál, Bálint Jezsó, Balázs Barátki, Dorottya Kövesdi, Szilvia Bősze, Ildikó Szabó, Tamás Felföldi, Erzsébet Oszwald, Parasuraman Padmanabhan, Balázs Zoltán Gulyás, Nazha Hamdani, Domokos Máthé, Zoltán Varga, Krisztián Szigeti

**Affiliations:** 1 Department of Biophysics and Radiation Biology, Semmelweis University, 37-47 Tűzoltó Street, Budapest, 1094 Hungary; 2 Institute for Radiopharmaceutical Cancer Research, Helmholtz-Zentrum Dresden-Rossendorf, 400 Bautzner Landstraße, 01328 Dresden, Germany; 3 Biological Nanochemistry Research Group, Institute of Materials and Environmental Chemistry, Research Centre for Natural Sciences, 2 Magyar Tudósok Körútja, Budapest, 1117 Hungary; 4 Doctoral School of Biology and Institute of Biology, Eötvös Loránd University, 1/C Pázmány Péter Sétány, Budapest, 1117 Hungary; 5 Department of Immunology, ELTE Eötvös Loránd University, 1/C Pázmány Péter Sétány, Budapest, 1117 Hungary; 6 MTA-ELTE Complement Research Group, Eötvös Loránd Research Network (ELKH), 1/A Pázmány Péter Sétány, Budapest, 1117 Hungary; 7 ELKH-ELTE Research Group of Peptide Chemistry, Eötvös L. Research Network, Eötvös L. University, 1/A Pázmány Péter Sétány, Budapest, 1117 Hungary; 8 Department of Microbiology, ELTE Eötvös Loránd University, 1/C Pázmány Péter Sétány, Budapest, 1117 Hungary; 9 Centre for Ecological Research, Institute of Aquatic Ecology, 29 Karolina Road, Budapest, 1113 Hungary; 10 Department of Anatomy, Histology, and Embryology, Semmelweis University, 58 Tűzoltó Street, Budapest, 1094 Hungary; 11 Lee Kong Chian School of Medicine, Nanyang Technological University, 11 Mandalay Road, Singapore, 30823 Singapore; 12 Cognitive Neuroimaging Centre, Nanyang Technological University, 59 Nanyang Drive, Singapore, 636921 Singapore; 13 Department of Cellular and Translational Physiology, Institute of Physiology, Ruhr University Bochum, 44801 Bochum, Germany; 14 HCEMM-Cardiovascular Research Group, Department of Pharmacology and Pharmacotherapy, University of Budapest, Budapest, 1089 Hungary; 15 CROmed Translational Research Centers, 37-47 Tűzoltó Street, Budapest, 1094 Hungary; 16 In Vivo Imaging Advanced Core Facility, Hungarian Center of Excellence for Molecular Medicine (HCEMM), 37-47 Tűzoltó Street, Budapest, 1094 Hungary

**Keywords:** Expression systems, Molecular engineering, Nanobiotechnology, Drug delivery, Bacteria, Vaccines

## Abstract

The important roles of bacterial outer membrane vesicles (OMVs) in various diseases and their emergence as a promising platform for vaccine development and targeted drug delivery necessitates the development of imaging techniques suitable for quantifying their biodistribution with high precision. To address this requirement, we aimed to develop an OMV specific radiolabeling technique for positron emission tomography (PET). A novel bacterial strain (*E. coli* BL21(DE3) *ΔnlpI, ΔlpxM*) was created for efficient OMV production, and OMVs were characterized using various methods. SpyCatcher was anchored to the OMV outer membrane using autotransporter-based surface display systems. Synthetic SpyTag-NODAGA conjugates were tested for OMV surface binding and ^64^Cu labeling efficiency. The final labeling protocol shows a radiochemical purity of 100% with a ~ 29% radiolabeling efficiency and excellent serum stability. The in vivo biodistribution of OMVs labeled with ^64^Cu was determined in mice using PET/MRI imaging which revealed that the biodistribution of radiolabeled OMVs in mice is characteristic of previously reported data with the highest organ uptakes corresponding to the liver and spleen 3, 6, and 12 h following intravenous administration. This novel method can serve as a basis for a general OMV radiolabeling scheme and could be used in vaccine- and drug-carrier development based on bioengineered OMVs.

## Introduction

Bacterial outer membrane vesicles (OMVs) are nano-sized extracellular vesicles (EVs) released by Gram-negative bacteria into their environment. Various mechanisms regarding their biogenesis have been proposed^1^. Their protein and lipid composition strongly resemble that of the outer membrane (OM) and the periplasmic space, however, some significant differences suggest the possibility that sorting mechanisms are involved in their formation^1,2^ too. Their roles in the life of bacteria and host-microbiome interactions are diverse, taking part in bacterial competition, biofilm formation, gene transfer, nutrient transport, antibiotic resistance, and stress response mechanisms while also serving as virulence factors^2–4^. Their contribution to the host’s immune homeostasis and their role in various diseases and disorders have been demonstrated^4^ making them prime candidates for diagnostics and even therapy.

OMVs are also emerging as a versatile vaccine platform due to their excellent adjuvant properties and ease of modification by genetic engineering, allowing researchers to express a wide array of foreign antigens on their surface^5,6^. Genetically engineered OMVs are a promising platform for targeted drug delivery applications, as demonstrated by their inherent tumor-targeting capabilities^7^ which can be further enhanced by introducing specific targeting molecules on them^8^. Their lumen can also be loaded with therapeutic compounds^9^.

Despite ongoing research interest in OMVs and their prospects in the pharmaceutical industry, our knowledge regarding their biodistribution is limited. Reports of their distribution have only been published in healthy animals and a few selected disease models^9–14^. It would be exciting to see how specific strain-based differences, mutations, or host pathologies affect the biodistribution of OMVs, however, quantifying their biodistribution is challenging. Methods based on fluorescent labeling and imaging with either whole-body fluorescence imaging devices or microscopy have been used previously^12^. These methods do not allow easy and precise quantification of biodistribution on the scale of organs or the whole animal, especially if one wishes to determine pharmacokinetic parameters. There have been some advances to address these challenges, including methods for optoacoustic imaging^15^ and nuclear medicine imaging, of which the latter can allow in vivo robust quantification of a radiolabeled compound’s concentrations at multiple time points in the same animal in 3D. Despite these clear advantages, OMV radiolabeling has only been reported in a few studies:

Pastor et al. presented a radiolabeling method^16^ based on the classic stannous-chloride reduction of technetium. Siddiqui et al.^17^ describe a method for the radiolabeling of bacteria and OMVs for positron emission tomography (PET) based on the bacterial expression of FyuA, an outer membrane receptor for the metallophore yersiniabactin (YbT). They demonstrated that ^64^Cu-labeled YbT can be incorporated into FyuA-expressing bacteria and their OMVs selectively. Zhe Li et al.^18^ report a deferoxamine-based ^89^Zr-labeling method of avian pathogenic *E. coli* OMVs.

In theory, there are many possible ways to approach OMV radiolabeling with different benefits and limitations. In this work we aimed to create an “imaging module” based on the widely used SpyCatcher-SpyTag^19^ system that enables stable, specific, and efficient radiolabeling, and allows the in vivo biodistribution study of OMVs. The outline of our proposed method is the following: a surface display system is used to anchor SpyCatcher to the OMV outer surface. SpyTag is fused with a molecule that can efficiently bind a radionuclide (e.g. a chelator). OMV radiolabeling is achieved by fusing the chelator-SpyTag to the SpyCatcher displayed on the OMV surface and directly labeling with the appropriate radionuclide. The most significant benefit of such an approach is its modularity: radiolabeling would be independent of bacterial strain, as any OMVs with SpyCatcher on their surface may be labeled using the same radiochemical procedure. The choice of chelator (or other radiolabeling target) could also be optimized for different radionuclides. Furthermore, the same method could be adapted to bacterial proteins genetically fused to SpyCatcher in addition to OMVs.

Surface display is a bioengineering technique that can be used to anchor polypeptides to the OM by inserting them into an extracellular region of an outer membrane protein. One such group of membrane proteins that are widely used for this purpose is autotransporters. Autotransporters, part of the type V secretion systems^20,21^ consist of three main regions: (i) an N-terminal signal sequence, responsible for Sec-dependent transport across the inner membrane, (ii) a passenger domain that determines the functional traits of the autotransporter and (iii) a C-terminal translocation unit that integrates into the OM allowing the translocation of the passenger domain through the membrane^22^. The passenger then can either stay attached to the rest of the autotransporter or dissociate after the cleavage of the polypeptide chain, depending on the type of autotransporter and its role. There are many subtypes of autotransporters with different structures and functions. In this study we compared two autotransporters to anchor SpyCatcher to the OMV surface: adhesin involved in diffuse adherence (AIDA-I)^23^ and haemoglobin binding protease (Hbp)^24–28^. Both belong to the Type Va (also called “classical”) autotransporter group, as indicated by having a monomeric structure and the release of their passenger domain following proteolytic cleavage, which is characteristic for this group (except for the EstA-like subgroup, where the passenger remains covalently bound^29^). Genetic engineering can be used to disrupt the cleavage site between the passenger and the translocation unit to anchor the passenger to the membrane, thus facilitating surface display.

SpyCatcher (SpC) is a protein that forms a spontaneous isopeptide bond with the peptide tag SpyTag (SpT)^19^. Derived from the modified CnaB2 domain of fibronectin-binding protein FbaB of *Streptococcus pyogenes*, this protein ligation system can be used to create covalent bonds between peptides and proteins both in vitro and in vivo^30,31^. The autotransporter passenger domain can be replaced partially or entirely with either one of the binding partners to create bacteria or OMVs that can bind the other binding partner^24,25,32^.

The combination of a surface display system with a protein ligation system enables the decoration of OMVs with proteins that could not be efficiently displayed by direct genetic fusion with the surface display system due to their large size or folding properties. It also makes the surface display of non-peptide molecules possible, therefore allowing the OM anchoring of a chelator for radiolabeling. Further combination with orthogonal protein ligation systems (e.g. SnoopCatcher/SnoopTag) can be used to create a modular platform allowing complex OMV surface functionalization^31^, making these systems promising for targeted drug delivery and vaccine development.

## Results and discussion

### Characterization of genetically engineered OMVs

We used OMVs isolated from a novel *Escherichia coli* strain (*E. coli* BL21(DE3) *ΔnlpI, ΔLpxM*, designated BL21.V) created with OMV production efficiency, heterologous protein expression capability, and reduced endotoxicity in mind. The base strain, BL21(DE3) was chosen for its favorable phenotype promoting its widespread application for protein expression. Particularly useful phenotypic traits are the absence of lon and OmpT proteases, of which the latter can greatly reduce surface display efficiency^33^. To counteract the possible hypovesiculating phenotype induced in some *E. coli* strains by OmpT deletion^34^, the *nlpI* gene was deleted using Lambda Red genome editing^35^. This deletion has been previously used to increase OMV production in *E. coli*^36^. Since our main goal was to develop a method for in vivo imaging we introduced another mutation aimed to ameliorate the endotoxic effects of *E. coli* OMVs^37,38^. For this we deleted the *lpxM* (*msbB*) gene to block the myristoylation of LPS creating a phenotype with lower endotoxicity^8,9,24^. The mutations were verified by sequencing the PCR products of the *nlpI* and *lpxM* gene regions.

OMV size distribution was determined using crude OMV samples (without purification). Briefly, bacteria were cultured in lysogeny broth at 37 °C for 16 h, then pelleted at 5000 g for 15 min using a fixed-angle rotor centrifuge. The supernatant was filtered with a 0.45 µm vacuum filter, then concentrated using a 100 kDa MWCO stirred cell ultrafiltration device and a tangential flow filter. Lastly, the concentrated supernatant was ultracentrifuged at 150,000 g for 2 h and resuspended in PBS and passed through a 0.45 µm centrifuge filter, resulting in a 1000 × concentration compared to the starting medium. Microfluidic resisitive pulse sensing (MRPS) and transmission electron microscopy was used for size distribution measurements.

**Figure 1:**
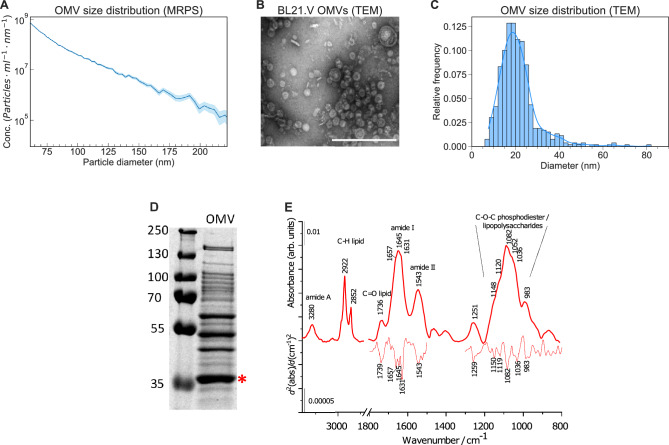
OMV characterization results. (**A**) Size distribution measured with MRPS shows an increasing number of vesicles with smaller diameters. (**B**) TEM photomicrograph of a crude OMV suspension. The scale bar represents 200 nm. (**C**) Size distribution of OMVs measured on TEM images. Bars represent the histogram; the solid blue line is the result of kernel density estimation. **D)** SDS-PAGE of crude OMV sample stained with PageBlue. Red asterisk indicates OmpF band. The uncropped gel image is presented in Fig. S11A. (**E**) IR spectrum of an OMV sample. To enhance the spectral information, second derivatives of selected wavenumber regions (amide I and amide II from 1800 to 1500 cm^−1^, and the fingerprint region from 1300 to 800 cm^−1^) are also shown.

MRPS resulted in 1.03 × 10^13^ ± 0.02 particles/ml using a microfluidic chip with a particle size detection range of 65–400 nm (Fig. 1A). The results show the tail of the OMV size distribution that decreases by multiple orders of magnitude in the measurement range indicating that larger particles are extremely rare in the sample. There was no peak in this range of the distribution, therefore to better characterize the size distribution under the MRPS cartridge detection limit we used TEM imaging. TEM images show the typical cup-shaped appearance of extracellular vesicles, (Fig. 1B) similar to previously reported *E. coli* OMVs^37,39,40^. After manually fitting ellipses around the vesicles and calculating the mean of minor and major axes, the results (Fig. 1C) reveal an average OMV diameter of 22.22 ± 9.02 nm, which is on the smaller side of previously reported OMV size ranges that typically fall within 10–300 nm^9,41–45^.

SDS-PAGE revealed a pattern similar to previously reported OMV isolates with characteristic OmpF bands^46–48^ (Fig. 1D).

After purification with size exclusion chromatography (SEC) using gravity columns filled with Sepharose CL-4B resin, we analyzed the samples using ATR-FTIR which is a fast, label-free method for studying molecular composition without perturbing the biological samples, so the characterization of intact OMVs is also possible (Fig. 1E). Characteristic bands of proteins and lipids can be identified in the spectrum. The bands around 3280, 1645, and 1543 cm^−1^ correspond to amide A, amide I, and amide II vibrations, respectively of the peptide backbone. The presence of lipid is confirmed by the methylene stretching of acyl chains at 2922 cm^−1^ and 2852 cm^−1^ and by the glycerol carbonyl stretching at 1736 cm^−1^ of the phospholipids. From the area of amide I (fitted by a Gaussian function) and the C–H stretching region (integrated from 3020 to 2800 cm^−1^) a spectroscopic protein-to-lipid ratio (P/L_spectr_) of 1.57 ± 0.09 was calculated, which is in line with our previous experiments on pure eukaryotic EVs (usually P/L_spectr_ falls between 0.5 and 2^49–52^) and has also been previously shown to be characteristic of EV quality^53,54^. Furthermore, the intensity of the amide I can be correlated to the protein concentration of EVs. Applying the protocol elaborated by Szentirmai et al.^50^, we obtained a total protein concentration of 0.63 ± 0.06 mg/ml for the purified OMVs. Unlike human blood-derived EVs, the fingerprint region of OMVs is dominated by strong bands between 1200 and 950 cm^−1^, assigned to C–O–C vibrations. Besides phosphodiesters, peptidoglycans and lipopolysaccharides, common on the bacterial membrane surface (affirmed by complex sugar vibrational bands at 1150, 1119, 1082, and 1036 cm^−1^) might have a contribution to the enhanced intensity of this spectral region^55^. It is worth mentioning the complexity of the amide I band, centered at 1645 cm^−1^; a similar spectral feature was observed in the IR spectrum of peptidoglycan films originating from *E.coli* bacteria^56^.

### Comparing surface display systems and SpyTag-chelator variants

The plasmids pET28-ASpC and pET28-HSpC were created by cloning the SpyCatcher gene into pAIDA1^23^ and pHbpD(Δd1)^24–28^, then cloning the resulting fusion genes to pET28 respectively. Their transcription is under the control of the T7 promoter enabling indirect control through the IPTG inducible expression of T7 RNA polymerase encoded in the BL21.V genome. Both autotransporters are frequently used for bacterial surface display and pHbpD(Δd1) was even used previously to display SpyCatcher on the surface of OMVs^23–28,33,57,58^. One notable difference between the two is that most of the AIDA-based display system’s passenger domain is replaced, anchoring SpyCatcher close to the OM^23^, while in the case of Hbp, only the d1 side-domain is replaced, leading to a larger distance between the OM and SpyCatcher^59^. Before OMV measurements we evaluated SpyCatcher surface expression on bacterial cells to optimize the induction protocol and growth conditions using carboxyfluorescein coupled SpyTag (SpT-CF) and flow cytometry. Based on these results we decided to use 40 µM IPTG for induction and 37 °C incubation temperature in lysogeny broth. For detailed description, results and discussion of these experiments see Supplementary results and Fig. S1–2.

**Figure 2:**
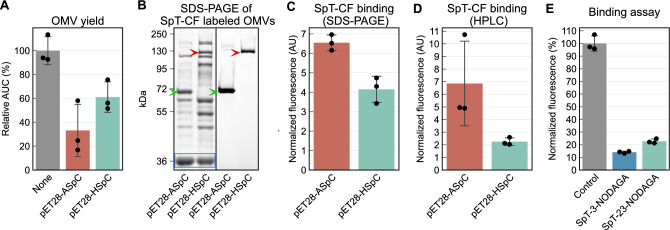
OMV yield and SpyTag binding. Bar charts represent mean ± SD. Swarm plots represent the individual measurements. (**A**) The OMV yields of bacteria harboring pET28-ASpC, pET28-HSpC or no plasmid were measured with SEC-HPLC. (**B**) SDS-PAGE of BL21.V OMV isolates labeled with SpT-CF. The left image shows the protein bands following PageBlue staining. The OmpF band used as loading control is highlighted with a blue rectangle. The right image shows SpT-CF fluorescence on the same gel. Green arrowhead: [AIDA-SpC]-[SpT-CF], red arrowhead: [HbpD-SpC]-[SpT-CF]. Uncropped gel images are presented in Fig. S11B and C. (**C**) SpT-CF binding of OMVs measured using SDS-PAGE. The fluorescence of specific bands was normalized to the OmpF band after PageBlue staining. (**D**) SpT-CF binding of the OMV isolates determined using HPLC. The AUC of the OMV peak of the fluorescent chromatogram was normalized to the AUC of the corresponding UV peak. (**E**) Binding assay results show that SpT-3-NODAGA can more efficiently block SpT-CF binding to TA-SpC OMVs than SpT-23-NODAGA.

We investigated whether the choice of surface display system could affect OMV yield. To avoid the influence of purification on the OMV concentration we used crude samples isolated in smaller batches. Since crude samples may contain soluble protein contaminants that affect protein quantification results, OMV yield was determined with size exclusion high performance liquid chromatography (SEC-HPLC) using the area under the first peak on the chromatogram obtained with a Sepharose CL-4B column. Before these experiments, we have thoroughly evaluated this resin for BL21.V OMVs and report our findings as Supplementary results and Fig. S3. The quantification results are summarized in Fig. 2A. A significant decrease in OMV yield was associated with bacteria harboring pET28-ASpC (AUC = 1.80 ± 0.210 AU for plasmidless vs 0.598 ± 0.395 AU for pET28-ASpC, two sample t-test *p* = 0.0095) and bacteria harboring pET28-HSpC (AUC = 1.1 ± 0.23 AU, two sample t-test *p* = 0.018). Although the difference in OMV yield between pET28-ASpC and pET28-HSpC harboring bacteria was not significant, pET28-ASpC shows lower OMV production. OMV yield being affected by autotransporter surface display has previously been reported in case of large constructs^28^. The exact mechanism is unknown.

SpyCatcher expression on the surface of OMVs isolated from bacteria harboring the two different plasmids was compared after incubating them with SpT-CF. Based on our preliminary experiments (Figure S4) we chose a 24 h incubation period and measured fluorescence using both SDS-PAGE and SEC-HPLC (Fig. 2B–D). SDS-PAGE revealed SpT-CF bands at the expected locations of AIDA-SpC and HbpD-SpC fusion proteins (65.896 kDa and 127.780 kDa respectively, Fig. 2B). Further bands most likely corresponding to proteolytic decay were also visible, especially in the case of pET28-HSpC. A double band around 130–150 kDa was also seen in the case of pET28-ASpC. This band was visible in SEC-purified OMV samples and was resistant to treatment with urea or trichloroacetate (TCA) and showed similar temperature-dependent mobility to AIDA-I^60^ (Figure S5). The exact origin of this band remains to be determined. SDS-PAGE data show that pET28-ASpC resulted in significantly higher SpyCatcher surface display on OMVs (6.546 ± 0.40 for pET28-ASpC and 4.152 ± 0.67 AU for pET28-HSpC, *p* = 0.0061, Fig. 2C), while HPLC data only reveal a non-significant difference (6.859 ± 3.35 AU for pET28-ASpC and 2.247 ± 0.30 AU for pET28-HSpC, *p* = 0.076, Fig. 2D). Based on these data, we decided to use pET28-ASpC for radiolabeling as the high levels of SpyCatcher on the OMV surface should allow higher specific activity. The negative effect on OMV yield can be overcome by isolating larger quantities.

Next, we created SpyTag-based bifunctional chelators for the radiolabeling of SpyCatcher expressing OMVs. We decided to conjugate the macrocyclic chelator NODAGA to SpyTag because it can chelate ^68^Ga or ^64^Cu (two widely used PET radionuclides) at mild reaction conditions with high specific activity and excellent stability^61,62^. Two different variants were synthetized: SpT-3-NODAGA and SpT-23-NODAGA. Both of them are based on a SpyTag peptide extended downstream and upstream according to the original sequence of fibronectin-binding protein^19^ labeled with NODAGA on either Lys^3^ (SpT-3-NODAGA) or Lys^23^ (SpT-23-NODAGA). The chelator was included in two different positions to investigate whether the macrocycle could interfere with SpyCatcher-SpyTag binding via steric hindrance. The inhibition of SpT-CF binding to OMVs isolated from pET28-ASpC harboring bacteria (TA-SpC OMVs) was significantly higher for SpT-3-NODAGA than SpT-23-NODAGA as measured using a simplified binding test (85.95% ± 0.85% and 77.28% ± 1.81% respectively, *p* = 0.006, Welch’s t-test) suggesting that C-terminal placement can lead to some amount of steric hindrance (Fig. 2E).

### Radiolabeling OMVs with ^64^Cu

**Figure 3:**
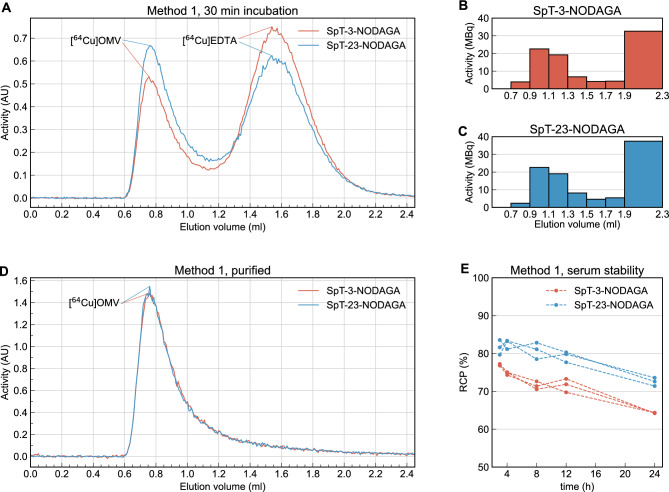
Radiolabeling results. (**A**) SEC-HPLC radiochromatogram of ^64^Cu labeled TA-SpC OMVs prepared by preincubating the vesicles with SpT-3/23-NODAGA. The first peak corresponds to ^64^Cu bound to the OMVs, while the second peak corresponds to free ^64^Cu chelated by EDTA. (**B**, **C**) Radiochromatograms of ^64^Cu-labeled OMVs obtained using a 2.1 ml gravity column packed with Sepharose CL-4B. The two fractions from 0.9–1.1 ml elution volume were pooled for further experiments. (**D**) SEC-HPLC radiochromatogram of the pooled fractions. (**E**) In vitro serum stability analysis of ^64^Cu-labeled OMVs.

Two approaches for OMV radiolabeling were tested. In one approach we first labeled the SpT-NODAGA variants with ^64^Cu, then incubated the SpC-expressing OMVs with the radiolabeled peptides. Although the peptide radiolabeling step was successful, leading to ~ 95% radiochemical purity (RCP) for both SpT-3-NODAGA and SpT-23-NODAGA, the reaction rate of SpyCatcher-SpyTag binding in the OMV incubation step proved to be too slow compared to the radioactive decay of ^64^Cu, so this approach was deemed infeasible. It is important to note that faster SpyCatcher-SpyTag variants^63^ could improve the performance of this approach. In the second method we first pre-incubated TA-SpC OMVs with either SpT-NODAGA variant for 24 h before carrying out the ^64^Cu radiolabeling. Radiochemical purity was determined using SEC-HPLC as the percentage of the area under the first (OMV containing) peak compared to the area under the entire radiochromatogram. This method resulted in 32.90% and 43.17% RCP for SpT-3-NODAGA and SpT-23-NODAGA labeled OMVs respectively (Fig. 3A). Samples were further purified using a Sepharose CL-4B gravity column and the two 200 µl fractions with the highest activity (0.9–1.3 ml elution volume) were pooled and analyzed with SEC-HPLC (Fig. 3B, C). SEC-HPLC analysis revealed only a single peak on the radio-chromatogram indicating 100% RCP (Fig. 3D). This method resulted in an overall radiolabeling efficiency of 29.20% and 28.87% for SpT-3-NODAGA and SpT-23-NODAGA labeled OMVs respectively. Serum stability was measured on samples incubated in FBS at 37 °C using SEC-HPLC and analyzed using multiple linear regression. The first measurements were done 3 h post-incubation, when the RCP values for SpT-3-NODAGA and SpT-23-NODAGA labeled OMVs were determined to be 77.08% ± 0.24% and 81.61% ± 1.95%, respectively. Statistical analysis revealed a significant decrease in serum stability from 3 to 24 h post-incubation amounting to a 0.51% decrease in RCP per hour (95% confidence interval: [0.43%, 0.58%]). Labeling with SpT-3-NODAGA also resulted in a significantly lower overall RCP compared to SpT-23-NODAGA (71.86 ± 4.45% and 79.36 ± 3.96% respectively, *p* = 0.0002) (Fig. 3E). These results show that although SpT-3-NODAGA can bind to the OMV surface more efficiently (as demonstrated using the SpT-CF binding assay), SpT-23-NODAGA leads to better ^64^Cu labeling results altogether. This suggests that the NODAGA macrocycle is more accessible for ^64^Cu at the SpyTag *C* terminal position when bound by SpyCatcher.

One additional observation we made is that even after longer incubations with EDTA, some ^64^Cu remains associated with OMVs. We suspect that this fraction of ^64^Cu is inside the OMV lumen and is slowly leaking out. The uptake of ^64^Cu into the vesicles should be possible through outer membrane porin OmpF^64^, which is the most abundant membrane protein of BL21.V OMVs as suggested by SDS-PAGE analysis. This could explain our serum stability results, where an RCP decrease of ~ 20% was visible in the first 3 h corresponding to intraluminal ^64^Cu. These serum stability results indicate that after an early release of a small fraction of ^64^Cu the labeled OMVs stay stable for up to 24 h.

### In vivo imaging of ^64^Cu-labeled OMVs

**Figure 4:**
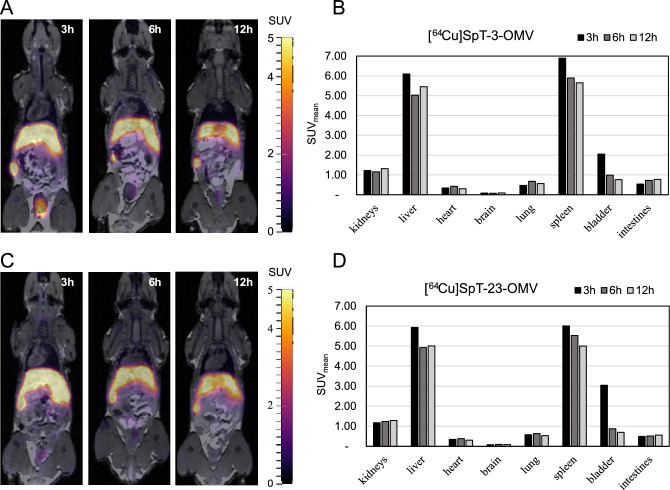
In vivo biodistribution of radiolabeled OMVs. (**A**) Representative slices of PET/MRI images taken 3, 6, and 12 h after the injection of OMVs labeled using SpT-3-NODAGA. (**B**) Decay-corrected mean standardized uptake values (SUV_mean_) of different organs of the same animal. (**C**) Representative slices of PET/MRI images taken 3, 6, and 12 h after the injection of OMVs labeled using SpT-23-NODAGA. (**D**) Decay-corrected mean standardized uptake values (SUV_mean_) of different organs of the same animal.

**Figure 5:**
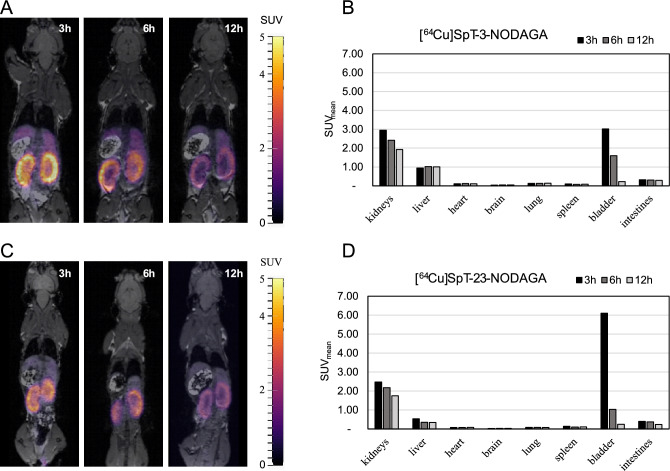
In vivo biodistribution of radiolabeled SpT-3/23-NODAGA. (**A**) Representative slices of PET/MRI images taken 3, 6, and 12 h after the injection of SpT-3-NODAGA labeled with ^64^Cu. (**B**) Decay-corrected mean standardized uptake values (SUV_mean_) of different organs of the same animal. (**C**) Representative slices of PET/MRI images taken 3, 6, and 12 h after the injection of SpT-23-NODAGA labeled with ^64^Cu. (**D**) Decay-corrected mean standardized uptake values (SUV_mean_) of different organs of the same animal.

The biodistributions of radiolabeled OMVs and SpT-3/23-NODAGA were determined in mice using PET/MRI at multiple time points (3, 6, and 12 h p.i.) following intravenous injection. One mouse was used per sample. We report the individual uptake of different organs (brain, lungs, heart, liver, kidneys, spleen, bladder, and intestines) in Table S2 for mean standardized uptake values (SUV_mean_) and Table S3 for uptake expressed as the percentage of organ activity to injected dose (%ID). Organ uptake expressed as %ID is also summarized in Figure S6. Our OMV biodistribution results summarized in Fig. 4 reveal that the choice of SpT-NODAGA variant for OMV labeling does not noticeably affect the measured vesicle distribution. OMV distribution is characterized by high uptake in the liver and spleen throughout the 12 h investigation interval. Even after 12 h, more than 40% of the injected dose was retained in the liver. The organs with the lowest vesicle uptake are the whole heart and brain. This pattern of biodistribution observed (the liver and spleen having the highest uptake) is similar to previously reported OMV biodistribution data^10,11^, however, a fair comparison is hard to make due to the differences in administration routes, imaging time, and imaging modality. To assess the in vivo stability of our radiolabeling approach we also carried out PET/MRI studies of [^64^Cu]SpT-3-NODAGA and [^64^Cu]SpT-23-NODAGA (Fig. 5). This is important, because if the radiolabeled peptides were to dissociate from the OMV surface their presence would affect the apparent distribution of OMVs. Fortunately, both peptides show early renal clearance with only 7.60% and 9.81% of the injected activity remaining in the animal 3 h p.i. of SpT-3-NODAGA and SpT-23-NODAGA respectively. The remaining activity further decreases to 3.82% and 2.89% at 12 h p.i. respectively. Furthermore, the large differences between the OMV and peptide distribution patterns, most notably the very low kidney uptake in the case of OMVs indicate that [^64^Cu]SpT-3/23-NODAGA stays associated with the OMVs for at least 12 h in vivo. There is also an observable difference between the biodistribution pattern of the two peptides: SpT-3-NODAGA shows higher liver uptake compared to SpT-23-NODAGA suggesting that the former might have higher clearance through the liver compared to the latter, however, more experiments are necessary to determine whether this difference is statistically significant.

Preclinical PET is a non-invasive high sensitivity and high resolution imaging method enabling the measurement of biodistribution data with high precision. This method has multiple advantages over optical imaging methods often used for EV biodistribution studies^65^. Although luminescence-based optical imaging has the advantage of enabling the investigation of biodistribution on a microscopic-cellular level, on the whole animal scale the light signal detected with these methods is subject to significant attenuation and scattering in the body imposing limitations on the experimental design. One such limitation is the choice of animal models used during the experiments. Using small rodents (mice) without fur (nude phenotype or removed before imaging) and investigating tissues/organs close to the surface can improve the quantifiability of these results. On the other hand, PET studies can be readily adapted to large animal models and can be translated to human medicine. The use of CT or MRI-based attenuation and scatter correction coupled with novel reconstruction methods make PET a quantitative imaging modality^66^. Our results could be converted to give the amount of OMVs (expressed in mg/ml protein) in a given volume, however, due to the lack of a standardized OMV protein quantification method we decided not to convert our biodistribution results. Based on our previous experience^50^, different protein quantification methods can give widely different results for the same EV samples. To investigate further, we have compared the Lowry assay (used in this study for protein quantification) with two other popular methods: the Bradford assay and the bicinchoninic acid assay (BCA) for OMV quantification. Our results suggest that although the protein contents determined by these methods can vary significantly, they are correlated and thus the choice of method doesn’t have a great influence when comparing samples measured with the same method (Figure S7). However, care must be taken when comparing OMV protein contents determined using different methods.

## Conclusion

Our results show that efficient surface display of SpyCatcher can serve as a basis for specific and stable OMV radiolabeling and quantitative molecular imaging. The versatility of our approach lies in its modular nature, as it consists of three main independent parts: the surface display system, the protein ligation system and the choice of chelator and radionuclide. Using this method as a template, various OMV-specific radiolabeling methods could be developed in the future by changing any of these parts to fit the specific requirements of the researcher. Our method could also be used as an “imaging module” for OMV based drug carrier and vaccine development, where bioengineered OMVs are often decorated with a protein ligation system into which the SpyCatcher-SpyTag-NODAGA system can be incorporated. Furthermore, our method could shed light on the distribution of OMVs originating from different bacterial strains residing in various body habitats and the change of this distribution in selected disease models, especially when studying the gut-brain axis or other gut-organ relationships.

## Materials and methods

### Peptide synthesis

Synthesis of Spytag (AHIVMVDAYKPTKGGGK) and its elongated (ATKGDAHIVMVDAYKPTKGSGGK) analogue peptide was carried out on Fmoc-Rink Amide MBHA resin (0.67 mmol/g) using Fmoc/tBu strategy. The synthetic SpyTag was further conjugated with carboxyfluorescein on its C-terminal lysine (resulting SpT-CF) and the elongated analogue was conjugated with NODAGA-NHS (Chematech, Dijon, France) on either Lys^3^ or Lys^23^ resulting in SpT-3-NODAGA and SpT-23-NODAGA respectively. For a detailed description see Supplementary Methods and Figure S8–S10.

### Culture conditions

For all bacterial liquid cultures lysogeny broth (LB) was used as a medium. LB was prepared by dissolving 25 g LB Broth (Miller) powder (Sigma Aldrich, USA) in 1 l of MilliQ water, adjusting the pH to 7.0 with NaOH, and autoclaving. LB agar was prepared by adding 1.5% Select agar powder (Sigma Aldrich, USA) to the LB medium and autoclaving. After autoclaving the LB agar was left to cool before adding antibiotics and pouring 20 ml into sterile culture plates (VWR, Germany). Ampicillin (SERVA Electrophoresis, Germany), kanamycin (SERVA Electrophoresis, Germany), or chloramphenicol (SERVA Electrophoresis, Germany) were used at concentrations of 100 µg/ml, 50 µg/ml, and 34 µg/ml respectively when necessary.

### Genome editing

Lambda Red genome editing was used to create two knockout mutations in *E. coli* BL21(DE3) cells (CMC0016, Sigma-Aldrich, USA) following the method described by Sheila Jensen and Alex Nielsen^67^. Briefly, the thermosensitive helper plasmid pSIJ8^35^ (plasmid number: #68,122, Addgene, USA) was electroporated into the BL21(DE3) cells. FRT-flanked kanamycin resistance cassettes with flanking regions homologous to the downstream and upstream ~ 50 bp regions of the *nlpI* and *lpxM* genes were PCR-amplified from Keio collection^68^ strains (Horizon discovery, UK, *nlpI* mutant: OEC4987-200828301, *lpxM* mutant: OEC4987-213605786) and used to carry out the gene deletions. The resulting double mutant BL21(DE3) *ΔnlpI, ΔlpxM*, designated BL21.V was used for all experiments. For more details, see Supplementary methods.

### Construction of surface display plasmids

The plasmids pAIDA1^23^ (plasmid number: #79180, Addgene, USA), pHbpD(Δd1)^24^ (a gift from Abera Bioscience), and pET28a were used to create the surface display systems for SpyCatcher surface expression using restriction cloning. First, SpyCatcher was cloned into pAIDA1 between the XbaI and SalI restriction sites, then the resulting AIDA-SpyCatcher fusion gene was inserted into pET28a resulting in the plasmid pET28-ASpC. SpyCatcher was also inserted between the SacI and BamHI restriction sites in pHbpD(Δd1) and the resulting HbpD-SpyCatcher fusion gene was inserted into pET28a to create the plasmid pET28-HSpC. *E. coli* BL21.V cells were transformed with the plasmids using electroporation. For more details, see Supplementary methods.

### Outer membrane vesicle isolation

We used two different OMV isolation protocols for different purposes. A small-scale isolation protocol was used to compare the OMV yield, SpyCatcher expression, and fluorescent SpyTag labeling of OMVs isolated from bacteria harboring different plasmids. For this protocol 40 ml LB medium in 250 ml flasks was inoculated with 0.32 ml overnight starter culture and grown at 37 °C, 180 RPM shaking until OD600 ≈ 0.7 was reached. At this point, 40 µM isopropyl β-d-1-thiogalactopyranoside (IPTG) was added and the culture was further incubated at 37 °C, 180 RPM shaking for 16 h. A volume of 30 ml of the culture was transferred to a 50 ml centrifuge tube and centrifuged at 5000 *g*, 15 min, and 4 °C to pellet bacterial cells. The supernatant was filtered using a 0.45 µm syringe filter (Millipore, USA) to remove remaining bacterial cells. An amount of 24.3 g of the filtered supernatant was loaded into a polycarbonate ultracentrifuge tube and centrifuged for 2 h at 150,000 *g*, 4 °C using an XL-80 ultracentrifuge (Beckman-Coulter, USA) equipped with a Type 50.2 Ti rotor. The pellet was resuspended in 250 µl PBS (137 mM NaCl, 2.7 mM KCl, 10 mM Na_2_HPO4, and 1.8 mM KH_2_PO_4_) and then filtered using a Costar Spin-X 0.45 µm centrifuge filter (Corning, USA).

A large-scale OMV isolation protocol was used for OMV characterization, to evaluate SpT-3/23-NODAGA binding and carry out the radiolabeling experiments. For large-scale OMV isolation, 2 × 250 ml LB medium in 2 L flasks was inoculated with 2 × 2 ml of overnight starter culture and grown at 37 °C, 180 RPM shaking for 16 h. IPTG induction was done at OD_600 _≈ 0.7 when necessary. The cultures were pooled and transferred to two 250 ml centrifuge bottles and centrifuged at 5000 *g*, 15 min, 4 °C. The supernatant was filtered using a Nalgene Rapid-Flow 500 ml bottle-top vacuum filter (0.45 µm, Thermo Scientific, USA) to remove bacteria. The filtrate was transferred to a stirred-cell ultrafiltration device (Millipore, USA) equipped with a 100 kDa NMW polyethersulfone ultrafiltration disc (Millipore, USA) and concentrated to ~ 60 ml. The volume of the concentrate was further reduced using a TFF-easy tangential filtration unit (Hansa Biomed, Estonia) to ~ 5 ml. After washing the concentrate with 60 ml PBS using the TFF-easy it was transferred to 24.3 ml polycarbonate ultracentrifuge tubes and centrifuged for 2 h at 150,000 *g*, 4 °C using an XL-80 ultracentrifuge (Beckman-Coulter, USA) equipped with a Type 50.2 Ti rotor. The pellet was resuspended in 500 µl PBS and filtered using a Costar Spin-X 0.45 µm centrifuge filter (Corning, USA). The OMV sample was then purified using a 2.1 ml size exclusion chromatography (SEC) column packed with Sepharose CL-4B (Cytiva, Germany).

Purified OMV samples were quantified according to their protein content measured with the Pierce modified Lowry protein assay kit (Thermo Scientific, USA) using a BSA standard and a BioTek Synergy 2 plate reader (BioTek, USA). OMV isolates were stored at 4 °C for up to 4 weeks.

### Characterization of outer membrane vesicles

BL21.V OMV size distribution was determined with MRPS and TEM photomicrograph analysis. The composition of OMVs was analyzed using ATR-FTIR. SDS-PAGE was used to determine protein composition and SpT-CF binding of OMV samples. For details see Supplementary methods.

### High-performance liquid chromatography (HPLC)

Peptides were analyzed using reversed-phase high-performance liquid chromatography (RP-HPLC), while OMV samples were analyzed using SEC-HPLC. The Jasco HPLC system was equipped with a PU-2089 pump unit, LC-NET II ADC, UV-2089 UV–Vis detector, Idex 7725i front-loading injector with a 100 μl loop, and a gamma-RAM Model 4 radio-HPLC detector (LabLogic, USA) equipped with a 25 µl cell for RP-HPLC and a 100 µl cell for SEC-HPLC. Fluorescent measurements were carried out on another JASCO HPLC system equipped with a PU-4180 pump, AS-4050 autosampler, UV-4075 UV–Vis detector, and an FP-2020 fluorescence detector controlled by ChromNAV Ver.2.

A Chromolith FastGradient RP-18e 50-2 mm column (Supelco, USA) was used for RP-HPLC measurements. MilliQ water with 0.1% trifluoroacetic acid (Solvent A) and 100% acetonitrile (Solvent B) were used as mobile phases. A sample volume of 1 µl was used and the gradient elution protocol was the following: 0–1 min: 100% Solvent A, 1–9 min: 0–80% Solvent B, 9–12 min: 80–0% Solvent B, 15–20 min: 100% Solvent A. A flow rate of 0.360 ml/min was used. UV absorbance was measured at 220 nm.

A Tricorn-5/50 column with a bed volume of ~ 1 ml (Cytiva, Germany) packed with Sepharose CL-4B (Cytiva, Germany) was used for SEC-HPLC. PBS (pH 7.4) was used as the mobile phase with a 0.5 ml/min flow rate amounting to a total elution time of 5 min per chromatogram. UV attenuation was measured at 280 nm. Fluorescence intensity was measured at 578 nm with 546 nm excitation.

Fluorescence and UV-attenuation chromatograms were split into two peaks manually to calculate areas under the curves. Due to their low signal-to-noise ratio, radio-chromatograms were exported and analyzed with curve fitting (see data analysis).

### SpyCatcher expression and binding assay with OMVs

SpyCatcher expression on the OMV surface was evaluated using SpT-CF for both plasmids. SpT-CF (2 mM) was diluted in the OMV samples to a final concentration of 10 µM and the mixture was incubated for 24 h at 4 °C on an orbital shaker. SDS-PAGE and SEC-HPLC were used to quantify the amount of OMV-bound SpT-CF. Measurements were carried out on 3 separate OMV isolates for each plasmid.

A simplified binding assay was used to compare the affinity of SpT-3-NODAGA and SpT-23-NODAGA to SpyCatcher expressing OMVs. A volume of 19 µl TA-SpC OMV (1.75 mg/ml) was incubated with 10 µM SpT-3/23-NODAGA (or MilliQ water for the negative control) at 4 °C on an orbital shaker. After 24 h, SpT-CF was added at a final concentration of 10 µM and the mixtures were further incubated for 24 h. Following incubation, SDS-PAGE was used to resolve specifically bound SpT-CF. The fluorescence intensity of the band corresponding to [AIDA-SpC]-[SpT-CF] was quantified and normalized to the density of the OmpF band measured after PageBlue staining. Measurements were carried out on 3 separate OMV isolates for each peptide.

### Radiolabeling and serum stability

TA-SpC OMVs (1.6 mg/ml) were incubated with 5 µM SpT-3/23-NODAGA for 24 h at 4 °C on an orbital shaker. Following incubation, free peptides were removed using a 2.1 ml Sepharose CL-4B gravity column equilibrated with sodium acetate buffer (0.1 M, pH = 6.0). A volume of 300 µl of the resulting OMV suspension was mixed with 142.5 ± 0.7 MBq ^64^CuCl_2_ (produced at the Helmholtz–Zentrum Dresden–Rossendorf, Germany, ~ 12 MBq/µl at the start of experiments) and adjusted to a final volume of 340 µl. The mixture was incubated for 20 min at 37 °C 300 RPM shaking. The reaction was stopped by adding 2 mM Na-EDTA to the mixture followed by 15 min incubation at room temperature. Radiochemical purity (RCP) was measured with SEC-HPLC. Free and EDTA-bound ^64^Cu was removed using a 2.1 ml Sepharose CL-4B column with PBS (pH = 7.4) as the equilibration buffer. Fractions (200 µl) were collected in individual tubes and radioactivity was measured using an ISOMED 2010 dose calibrator (Nuvia, France). The two fractions with the highest activity were pooled. For serum stability measurements 20 µl of the pooled OMV sample was mixed with 80 µl fetal bovine serum (Thermo Fisher, USA) and incubated at 37 °C 300 RPM shaking. The samples were analyzed with SEC-HPLC at 3, 4, 8, 12 and 24 h post-incubation.

### In vivo imaging

Four healthy 21-week-old male BALB/c mice (body weight = 24.73 ± 3.88 g) bred in the Animal House of Semmelweis University were used for the biodistribution studies. Animals were allowed free access to food and water and were kept under humidity, temperature, and light-controlled conditions. All procedures were conducted by the ARRIVE guidelines and the guidelines set by the European Communities Council Directive (86/609 EEC) and approved by the Animal Care and Use Committee of the IEM and Semmelweis University (PE/EA/929–5/2021). A volume of 120 μl radiolabeled OMV suspension with an activity of 10.17 ± 1.10 MBq (Mean ± SD) was administered intravenously into the lateral tail vein. Mice were anesthetized with isoflurane (3.5–4% induction, then reduced to 1.5% for the maintenance of anesthesia during imaging) for the whole duration of imaging. PET/MRI acquisitions were carried out using a nanoScan PET/MRI 3T (Mediso, Hungary) equipped with a Mediso mouse whole-body coil resulting in a 3D PET resolution of 1.4 mm at full width at half maximum (FWHM) and a PET sensitivity of 200 true detection events per second /kBq. A GRE 3D sequence with a 45° flip angle, 15 ms repetition time, and 4.2 ms echo time with 2 excitations averaged was used to acquire 64 coronal slices with a slice thickness of 0.4 mm and an in-plane resolution of 0.33 mm. PET images were acquired 3 h, 6 h, and 12 h post-injection (p.i.) from the 400–600 keV energy window using an acquisition time of 5 min and a 5 ns coincidence time window. The Tera-Tomo 3D (Mediso, Hungary) algorithm with MRI-based attenuation and scatter correction, normal regularization, median and spike filter, and edge-artifact reduction was used to reconstruct the images with 2 iterations and 6 subsets resulting in 0.6 mm isovoxel size. Images were analyzed using vivoquant 1.22 (inviCRO, US). Volumes of interest (VOI) were manually delineated around selected organs (brain, lungs, heart, liver, spleen, kidneys, bladder, and intestines). VOI uptake data are reported in mean standardized uptake values (SUVmean) and percentage of injected dose. 3D Slicer 4.11^69^ was used to create figures for illustration.

### Data analysis

Microsoft Excel and Python 3.7.12 was used to process and analyze most data and plot graphs. The Python package SciPy^70^ was used for statistical hypothesis tests.

SEC-HPLC radiochromatograms were zeroed by subtracting the mean of the 0.25–0.75 min interval. Exponentially modified Gaussian (EMG) and general exponentially modified Gaussian (GEMG) functions^71^ were used as peak models. In the case of radio-chromatograms, radiochemical purity was calculated as the percentage of the area under the first peak (corresponding to OMVs).

A linear mixed-effects model with random intercepts (for each sample) in R (v. 4.1.1.)^72^ using the package nlme^73^ was used to analyze serum stability data.

Numeric results are presented as mean ± standard deviation when applicable unless otherwise noted.

### Ethics approval

All animal experiments have been approved by the Animal Care and Use Committee of the IEM and Semmelweis University (PE/EA/929-5/2021).

### Supplementary Information

Supplementary Information.

## Data Availability

The datasets used and/or analysed during the current study are available from the corresponding author on reasonable request.

## Abbreviations

AIDA: Adhesin involved in diffuse adherence
ATR-FTIR: Attenuated total reflectance-Fourier transform infrared spectroscopy
BCA: Bicinchoninic acid assay
BSA: Bovine serum albumin
CF: Carboxyfluorescein
CT: Computed tomography
EDTA: Ethylenediaminetetraacetic acid
EMG: Exponentially modified Gaussian
EV: Extracellular vesicle
FBS: Fetal bovine serum
FRT: Flippase recognition target
FWHM: Full width at half maximum
GEMG: General exponentially modified Gaussian
HPLC: High-performance liquid chromatography
Hbp: Haemoglobin binding protease
ID: Injected dose
IR: Infrared
LB: Lysogeny broth
LPS: Lipopolysaccharide
MRI: Magnetic resonance imaging
MRPS: Microfluidic resistive pulse sensing
NMW: Nominal molecular weight
NODAGA: 2-[1,4,7-Triazacyclononan-1-yl-4,7-bis(tBu-ester)]-1,5-pentanedioic acid
OD600: Optical density at 600 nm
OM: Outer membrane
OMV: Outer membrane vesicle
OmpF: Outer membrane porin f
PBS: Phosphate-buffered saline
PCR: Polymerase chain reaction
PET: Positron emission tomography
RCP: Radiochemical purity
RP-HPLC: Reversed-phase high-performance liquid chromatography
RPM: Revolutions per minute
SDS-PAGE: Sodium dodecyl sulfate polyacrylamide gel electrophoresis
SEC: Size-exclusion chromatography
SEC-HPLC: Size-exclusion high-performance liquid chromatography
SUV: Standardized uptake value
SpC: Spycatcher
SpT: Spytag
TA-SpC: Designation of OMV samples isolated from bacteria harboring pet28-aspc
TEM: Transmission electron microscopy
TFF: Tangential flow filtration
VOI: Volume of interest
