# Supplementary material for: Molecular imaging of bacterial outer membrane vesicles based on bacterial surface display
Source: Sci Rep. 2023 Oct 31;13:18752. doi: 10.1038/s41598-023-45628-9 (PMC10618197; doi:10.1038/s41598-023-45628-9)
Supplement: Supplementary file 1 — Supplementary Information. [file 41598_2023_45628_MOESM1_ESM.docx]

Molecular imaging of bacterial outer membrane vesicles based on bacterial surface display

Dávid Szöllősi^1^, Polett Hajdrik^1^, Hedvig Tordai^1^, Ildikó Horváth^1^, Dániel S. Veres^1^, Bernadett Gillich^1^, Kanni Das Shailaja^1^, László Smeller^1^, Ralf Bergmann^1,2^, Michael Bachmann^2^, Judith Mihály^3^, Anikó Gaál^3^, Bálint Jezsó^3,4^, Balázs Barátki^5^, Dorottya Kövesdi^5,6^, Szilvia Bősze^7^, Ildikó Szabó^7^, Tamás Felföldi^8,9^, Erzsébet Oszwald^10^, Parasuraman Padmanabhan^11,12^, Balázs Zoltán Gulyás^11,12^, Nazha Hamdani^13,14^, Domokos Máthé**^†^** ^1,15,16^, Zoltán Varga**^†^** ^1,3^, Krisztián Szigeti***^†^** ^1^

1: Department of Biophysics and Radiation Biology, Semmelweis University, 37-47 Tűzoltó Street, Budapest 1094, Hungary

2: Institute for Radiopharmaceutical Cancer Research, Helmholtz-Zentrum Dresden-Rossendorf, 400 Bautzner Landstraße, Dresden 01328, Germany

3: Biological Nanochemistry Research Group, Research Centre for Natural Sciences, Institute of Materials and Environmental Chemistry, 2 Magyar Tudósok Körútja, Budapest 1117, Hungary

4: Doctoral School of Biology and Institute of Biology, Eötvös Loránd University, 1/C Pázmány Péter Sétány, Budapest 1117, Hungary

5: Department of Immunology, ELTE Eötvös Loránd University, 1/C Pázmány Péter Sétány, Budapest 1117, Hungary

6: MTA-ELTE Complement Research Group, Eötvös Loránd Research Network (ELKH), 1/A Pázmány Péter Sétány, Budapest 1117, Hungary

7: ELKH-ELTE Research Group of Peptide Chemistry, Eötvös L. Research Network, Eötvös L. University, 1/A Pázmány Péter Sétány, Budapest 1117, Hungary

8: Department of Microbiology, ELTE Eötvös Loránd University, 1/C Pázmány Péter Sétány, Budapest 1117, Hungary

9: Institute of Aquatic Ecology, Centre for Ecological Research, 29 Karolina Road, Budapest 1113, Hungary

10: Department of Anatomy, Histology, and Embryology, Semmelweis University, 58 Tűzoltó Street, Budapest 1094, Hungary.

11: Lee Kong Chian School of Medicine, Nanyang Technological University, 11 Mandalay Road, Singapore 30823, Singapore.

12: Cognitive Neuroimaging Centre, Nanyang Technological University, 59 Nanyang Drive, Singapore 636921, Singapore.

13: Department of Cellular and Translational Physiology, Institute of Physiology, Ruhr University Bochum, 44801 Bochum, Germany

14: HCEMM-Cardiovascular Research Group, Department of Pharmacology and Pharmacotherapy, University of Budapest, HU-1089 Budapest, Hungary.

15: CROmed Translational Research Centers, 37-47 Tűzoltó Street, Budapest 1094, Hungary

16: In Vivo Imaging Advanced Core Facility, Hungarian Center of Excellence for Molecular Medicine (HCEMM), 37-47 Tűzoltó Street, Budapest 1094, Hungary

* corresponding author: Krisztián Szigeti

**†** authors contributed equally

Supporting information

# Supplementary Results

## Optimization of SpyCatcher surface expression

To optimize SpyCatcher surface display on bacterial cells harboring either pET28-ASpC or pET28-HSpC, multiple IPTG concentrations (40 µM, 400 µM and 2000 µM) combined with different growth temperatures (24 °C and 37 °C) were tested. Cells were labeled with carboxyfluorescein-conjugated SpyTag (SpT-CF) 16 h post-induction and measured with flow cytometry (FCM). FCM measurements showed comparable SpyCatcher expression at 24 °C, pET28-HSpC performing better at 37 °C (Figure S1). Leaky expression was observable in the case of both plasmids, especially at 24 °C where increasing IPTG concentrations lead to lower median expression. Increasing IPTG concentrations also lead to the separation of two distinct populations along the SSC axis, most likely corresponding to cells with different protein translation activities. Cells belonging to these distinct populations also had different fluorescence intensity distributions with higher intensities at higher SSC values (Figure S2). Leaky expression of membrane proteins from the T7 promoter in E. coli BL21(DE3) grown in LB has previously been investigated by Zhang et al. [1]. The authors found that the addition of IPTG can lead to the increase of non-producing cell populations ultimately leading to decreased membrane protein expression efficiency compared to cells with no IPTG added. This is mostly in agreement with our FCM results where a decrease in median expression and the appearance of different cell populations can be observed for increasing IPTG concentrations in multiple conditions. Based on our results this decrease in expression can be avoided by using a low IPTG concentration (40 µM) combined with a growth temperature of 37 °C. This IPTG concentration is ten times lower than the one investigated by Zhang et al. [1].

## Evaluation of CL-4B resin for OMV analysis and purification

To demonstrate that Sepharose CL-4B, the resin used throughout this study for both analytic HPLC and SEC purification, can efficiently separate OMVs from protein impurities we have compared OMV samples before and after SEC purification using SDS-PAGE, TEM and ATR-FTIR spectroscopy. SDS-PAGE results demonstrate that the relative intensity of the OmpF band is increased in the sample following SEC purification. (Figure S3. A) The reduction of soluble contaminants is also evident on TEM photomicrographs stained with uranyl-acetate: SEC purification greatly reduces the substantial background staining of crude OMV isolates that is most likely due to soluble proteins adsorbed to the grid (Figure S3. B, C). The comparison of the OMV samples’ IR spectra before and after purification (Figure S3. D) reveals a higher amount of protein, reflected by the higher relative intensity of amide A, amide I and amide II bands at 3280, 1646 and 1539 cm^-1^, respectively, and those belonging to side amino chains around 1400 cm^-1^. This spectral change is quantified by the P/L_spectr_ value: 2.33± 0.05 for a crude OMV sample and 1.57± 0.09 after SEC purification. As to the fingerprint spectral region, some alterations are witnessed, too. For the crude OMV sample, bands at 1240, 1068, and 1038 cm^-1^ are dominating the low wavenumber part of the spectrum. The bands at 1240 and 1068 cm^-1^ might belong to the phosphate vibrations of cell membrane phospholipids or DNA, while the strong band at 1036 cm^-1^ is related to sugar modes of peptidoglycans [2]. After purification, the intensity of these bands is also suppressed. By eluting OMVs isolated from pET28-ASpC harboring bacteria in fractions and labeling the fractions with SpT-CF we also found that most of the OMVs are eluted early, forming a single peak (Figure S3. E). Altogether these findings support the use of Sepharose CL-4B for the purification and HPLC analysis of OMVs. An important note is that the UV signal of nano-sized vesicles is partially caused by scattering, thus it can be size-dependent. Therefore, the size distribution of vesicles of different organisms should be evaluated before a comparison is made based on HPLC.

# Supplementary methods

## Peptide synthesis

The Amino acid derivatives and Rink Amide 4-methylbenzhydrylamine (Rink Amide MBHA) resin were purchased from IRIS Biotech GmbH (Marktredwitz, Germany). Reagents for synthesis: N,N’-diisopropylcarbodiimide (DIC), N-diisopropyl-ethylamine (DIEA), 1-hydroxybenzotriazole (HOBt), 1,8-diazabicyclo-[5.4.0]undec-7-ene (DBU), piperidine, hydrazine hydrate (N_2_H_2_ hydrate), triisopropylsilane (TIS), 5(6)-carboxyfluorescein (CF) were purchased from Sigma Aldrich (Hungary). Trifluoroacetic acid (TFA) and solvents for the synthesis as well as HPLC grade acetonitrile were obtained from Molar Chemicals (Hungary).

Synthesis of Spytag (AHIVMVDAYKPTKGGGK) and its elongated (ATKGDAHIVMVDAYKPTKGSGGK) analogue peptide was carried out on Fmoc-Rink Amide MBHA resin (0.67 mmol/g) using Fmoc/tBu strategy. For couplings carbodiimide (DIC)/HOBt procedure was applied. The N-termini of the peptides was acetylated with Ac_2_O/DIEA/DMF (1 : 1 : 3, v/v/v) mixture (reaction time: 30 min). To synthesize fluorescent Syptag peptide derivative (SpT-CF), orthogonally protected lysines were used; the C-terminal lysine residue was protected with Dde, and the other lysines were protected with Boc protecting groups. After acetylation, Dde protecting group was removed with 2% N_2_H_2_ hydrate in DMF for 6x2 min. After washing with DMF, the peptide was labelled with 5(6)-carboxyfluorescein/DIC/HOBt (2.5:5:5 equiv) for 60 min.

The elongated Spytag analogue contains four lysines, three of them were protected with Dde protecting group, and the fourth one (either ^3^K or ^23^K) was Boc-protected amino acid derivative. The peptides were obtained by cleavage from the resin with TFA containing D.I. water and triisopropyl silane (95:2.5:2.5) as scavengers. By this cleavage procedure, side-chain protecting groups, except Dde from lysine residues (elongated Spytag analogues), were also removed. Crude products were precipitated by dry diethyl-ether, dissolved in distilled water-acetonitrile mixture and freeze-dried. The crude peptides were characterized by analytical RP-HPLC and MS.

SpT-3-NODAGA and SpT-23-NODAGA were obtained by NODAGA labeling of elongated Spytag variants (with either Boc-protected ^3^K or ^23^K, respectively) were carried out in the liquid phase without purification. The crude, Dde-protected peptides were dissolved in DMF (5 µM/mL) and were reacted with 1 equiv NODAGA-NHS (Chematech, Dijon, France) and DIEA (20 equiv). The reaction mixture was stirred at 0°C for 15 min, and after at RT for 24 h. At the end of the reaction, 2% N_2_H_2_ hydrate was added to the reaction mixture to cleave the Dde protecting groups from the lysine residues. The Dde cleavage procedure was carried out in RT for 60 min, while white precipitation occurred. The reaction mixture was diluted with eluent

The crude products were purified immediately on a Thermo Fisher Scientific Dionex Ultimate 3000 HPLC system (Waltham, Massachusetts, USA), using a semipreparative a Phenomenex Jupiter Proteo C12 column (250x10mm I.D.) with 10 µm silica (GL Sciences, Tokyo, Japan). The flow rate was 4 ml/min. Linear gradient elution was applied.

The identification of the products was achieved by mass spectrometry. The MS analysis was performed on Thermo Scientific Q Exactive Focus Hybrid Quadrupole-Orbitrap Mass Spectrometer. Samples were dissolved in 50% acetonitrile - 50% water containing 0.1% acetic acid. Mass spectra were recorded in a positive mode in the m/z 50-2000 range (Figure S9, S10, S11. A). The purity of the products was determined using RP-HPLC as described below (see section entitled “High-performance liquid chromatography”). Results are shown in Figure S9, S10, S11. B.

Genome editing

pSIJ8 [3] (plasmid number: #68122, Addgene, USA) was electroporated into the BL21(DE3) cells with a GenePulser Xcell Electroporation System (Bio-Rad, USA) using a 1 mm electroporation cuvette (Bio-Rad, USA). A time-constant protocol was used with 1.8 kV voltage and 5 ms time-constant. All further electroporations were done using the same setup. Cells harboring pSIJ8 were grown in LB medium containing 100 µg/ml ampicillin at 30 °C. FRT-flanked kanamycin resistance cassettes were amplified using Q5 High-fidelity DNA polymerase (New England BioLabs, USA) with primer pairs nlpI-fw, nlpI-rv; and lpxM-fw, lpxM-rv (Supplementary Table 1) respectively. The primers were synthetized by Integrated DNA Technologies (USA). To separate the product from the template and the primers agarose gel-electrophoresis using a 1% Topvision agarose gel (Thermo scientific, USA) was carried out. Gel-excision and Nucleospin PCR cleanup kit (Macherey Nagel, Germany) was used to purify the PCR products.

The lambda-Red genes encoded on pSIJ8 were induced with 20 mM L-(+)-arabinose (Sigma-Aldrich, USA) at OD_600_ ~0.4 to facilitate recombination. Cells were grown for another 45 minutes, then made electrocompetent following the protocol described in [4]. A volume of 5 µl of the PCR product with *nlpI* homologous regions was used to transform 50 µl of electrocompetent cells. After electroporation with the PCR products followed by recovery in LB medium, cells were grown on LB plates containing 100 µg/ml ampicillin and 50 µg/ml kanamycin at 30 °C. Colony PCR using Q5 polymerase and the appropriate primer pairs was used to verify successful recombination. A 2-hour culture grown in 1 ml LB with ampicillin and kanamycin was centrifuged at 6500 g, 2 min, RT, resuspending in 1 ml LB with ampicillin, and treated with 50 mM L-rhamnose (Sigma-Aldrich, USA) for 4 hours to delete the FRT-flanked kanamycin resistance cassette. Next, cells were grown on LB plates containing 100 µg/ml ampicillin. To delete the *lpxM* gene from the resulting BL21(DE3) *ΔnlpI* strain, the same procedure was repeated using the PCR product with *lpxM* homologous regions. To remove pSIJ8, cells were grown at 37 °C in LB, then spread on two LB plates - one containing ampicillin - and grown at 37 °C to verify successful removal of the plasmid. Both deletions were verified by PCR and the presence of the remaining FRT scar sequence was verified by sequencing the PCR products.

Construction of surface display plasmids

All enzymes and buffers used for the restriction cloning procedures were products of Thermo Scientific (USA) unless indicated otherwise. Q5 High-fidelity DNA polymerase (New England BioLabs, USA) was used to amplify the inserts and DreamTaq DNA polymerase was used for colony PCR’s following the manufacturers’ instructions. A Nucleospin gel and PCR clean-up kit (Macherey-Nagel, Germany) were used to purify PCR products and a Qiaprep spin miniprep kit was used to prepare plasmids from 10 ml overnight bacterial liquid cultures before restriction cloning. The purity and concentration of DNA samples were determined using a NanoDrop 1000 spectrophotometer (Thermo Scientific, USA). All primer sequences can be found in Supplementary Table 1. The resulting plasmid sequences were verified with sequencing.

SpyCatcher was PCR amplified from a synthetic template (Supplementary Table S1.) created by Cyagen Biosciences (Germany) with primer pairs SpC_AIDA_fw, SpC_AIDA_rv; and SpC_Hbp_fw, SpC_Hbp_rv for insertion into pAIDA1 (plasmid number: #79180, Addgene, US) and pHbpD(Δd1) respectively. For pAIDA1 both the inserts and the plasmid were digested using XbaI and SalI restriction enzymes in 2x Tango buffer for 1h at 37 °C. For pHbpD(Δd1) the plasmid and the inserts were digested using SacI and BamHI in 1x Buffer BamHI for 1h at 37 °C. Plasmids were dephosphorylated using FastAP thermosensitive alkaline phosphatase for 10 min at 37 °C following manufacturer’s instructions. The reaction was stopped by incubating the mixture for 5 min at 75 °C. After clean-up ~100 ng of the plasmids and 5x molar excess of the corresponding inserts were mixed and incubated with T4 DNA Ligase (1 U) in 1x T4 DNA Ligase Buffer following manufacturer’s instructions.

The final plasmids pET28-ASpC and pET28-HSpC were created similarly. The Hbp-SpyCatcher fusion gene was amplified from pHbpD-SpC using the primer pair HSpC_fw and HSpC_rv. Following PCR clean-up the product was digested using PciI and NheI while pET28a was digested with NcoI and NheI in 1x Tango buffer. The AIDA-SpyCatcher fusion gene was amplified from pAIDA-SpC using the primer pair ASpC_fw and ASpC_rv. Following PCR clean-up the product was digested using PciI and SacI while pET28a was digested with NcoI and SacI in 1x Tango buffer. Dephosphorylation and ligation were carried out as described above.

The resulting plasmids were used to transform chemically competent NEB5-alpha cells (New England Biolabs, USA) following the manufacturer’s instructions. Glycerol stocks were prepared from the transformed cells and stored at -80 °C before use.

## Characterization of outer membrane vesicles

MRPS measurements were carried out using an nCS1 particle size analyzer (Spectradyne, USA). Bovine serum albumin (Sigma, USA) was dissolved in PBS to a final concentration of 1 mg/ml and filtered using a 100 kDa MWCO Amicon Ultra 0.5 ml centrifuge filter (Merck, USA) by centrifuging at 10 000 g, 5 min. OMV samples were 1000-fold diluted in the BSA solution before measurement. A volume of 4 µl of the dilute sample was measured using a TS-400 cartridge (Spectradyne, USA) with a nominal particle diameter range of 65-400 nm. Traceable polystyrene (PS) bead with a 90 nm nominal diameter was used for calibration (PS90, 1 w/v%, Polysciences Inc., USA). The stock solution of the particle standard was diluted to 4000-fold in 1 mg/mL filtered BSA solution.

For transmission electron microscopy, a 2 µl OMV sample was applied to a Lacey Carbon Type-A copper grid (Ted Pella Inc, USA) and incubated for 2 minutes. The excess suspension was blotted with a piece of filter paper. Next, the grids were placed on a drop of 2% uranyl acetate (v/v) solution for 1 min, blotted, then placed on another drop again for 1 min. After blotting the excess uranyl acetate solution, the grids were air-dried and viewed using a JEOL JEM-1011 TEM (JEOL, Peabody, MA, USA) operated at 80 kV. The camera used for image acquisition was a Morada TEM 11 MPixel from Olympus (Olympus, Tokyo, Japan) using iTEM5.1 software for metadata analysis. OMV size distribution was determined using NIH ImageJ software by manually fitting ellipses around all vesicles. All round or slightly elongated objects with well-defined smooth edges on 3 photomicrographs were included in the analysis. A total of 529 OMVs were measured this way. The means of the major and minor axis lengths were calculated and a histogram was created using python.

The infrared spectra were recorded with a Bruker Vertex80v (Bruker Optics, Billerica, MA) FTIR spectrometer equipped with a high sensitivity MCT (mercury-cadmium-telluride) detector. Each spectrum was collected by averaging 128 scans at 2 cm^-1^ resolution. The sample (3 µl) was dried on a single reflection diamond ATR crystal. Spectral manipulations were performed using the GRAMS/32 software package (Galactic Inc, USA).

## Sodium dodecyl sulfate-polyacrylamide gel electrophoresis (SDS-PAGE)

The 10% resolving polyacrylamide gels were prepared in a Mini-Protean Tetra casting stand (Bio-Rad, USA) with a 1 mm spacer. A 15-well comb was used (mini-PROTEAN, BioRad) for the 5% stacking gel. Samples were mixed 1:1 with 2x reducing sample buffer (50 mM Tris-PO4, pH 6.8; 2% SDS; 2 mM EDTA (Sigma Aldrich, USA); 20% glycerol (Sigma Aldrich, USA); 0.02% bromophenol blue (Sigma Aldrich, USA); 10% 2-mercaptoethanol (Sigma Aldrich, USA) and incubated at 95 °C for 5 minutes unless specified otherwise. A volume of 20 µl sample or 5 µl of prestained protein ladder (PageRuler, Thermo Scientific) was loaded into the wells of the stacking gel. For fluorescent OMV samples labeled with SpT-CF, the gel was washed 3x with ultrapure water following electrophoresis and scanned using an Amersham Typhoon 9400 (GE, USA). Gels were stained with PageBlue protein staining solution (Thermo Scientific, USA) according to the manufacturer's instructions. A GelLogic 212 Pro (Carestream, USA) system was used to image the PageBlue stained gels.

## Flow cytometry (FCM)

Liquid cultures of BL21.V (empty, or harboring surface display plasmids) were grown in LB supplemented with the necessary antibiotics at 37 °C, 180 RPM shaking until OD600 ~0.7, divided into 5 ml batches and IPTG (0, 40, 400, 2000 µM) was added to induce protein expression. Following the addition of IPTG, the cultures were incubated for 16 hours at 24 °C or 37 °C shaking at 180 RPM. Final OD600 was measured with a NanoDrop 1000 spectrophotometer (Thermo Scientific, USA). Depending on the OD600 values, a volume of 80-200 µl of the culture was diluted in 800 µl PBS and centrifuged at 5000 g, 4°C for 10 min. The supernatant was removed, then the pellets were resuspended in PBS containing 10 µM SpT-CF and incubated for 1 h at room temperature on an orbital shaker. Next, 800 µl PBS was added and the samples were centrifuged at 5000 g, 4°C for 10 min. After removing the supernatant, the pellets were resuspended in 800 µl PBS. This washing procedure was repeated two additional times. The pellets were finally resuspended in 400 µl PBS and transferred to round bottom plastic tubes (Sarstedt, Germany). FCM measurements were carried out using a FACSCalibur (BD Biosciences, USA) flow cytometer. FlowJo v. 10 (BD biosciences, USA) was used to define gates for E. coliE. coli on a log-log forward scatter/side scatter plot. Fluorescence data measured on the FL1 channel was exported as CSV files and loaded into python using pandas [35] and plotted using seaborn [36] and matplotlib [37]. A Kruskal-Wallis test with Dunn’s post-hoc test was used to compare the fluorescence of samples from the selected induction protocol (40 µM IPTG, 37 °C).

Data analysis

HPLC peaks were fitted using an exponentially modified Gaussian (EMG) and general exponentially modified Gaussian (GEMG):

| $\boldsymbol{GEMG}\left( \boldsymbol{t} \right)\boldsymbol{=}\frac{\boldsymbol{A}}{\boldsymbol{1+b}}\left( \boldsymbol{EMG}_{\boldsymbol{1}}\boldsymbol{+b}\boldsymbol{EMG}_{\boldsymbol{2}} \right)$ | Eq. 1. |
| --- | --- |

Where

| $\boldsymbol{EMG}_{\boldsymbol{i}}\left( \boldsymbol{t} \right)\boldsymbol{=}\boldsymbol{e}^{\boldsymbol{q}_{\boldsymbol{i}}}\boldsymbol{I}_{\boldsymbol{i}}\boldsymbol{/}\boldsymbol{\tau}_{\boldsymbol{i}}$ | Eq. 2. |
| --- | --- |
| $\boldsymbol{q}_{\boldsymbol{i}}\boldsymbol{=}\frac{\boldsymbol{\sigma}^{\boldsymbol{2}}}{\boldsymbol{2}\boldsymbol{\tau}_{\boldsymbol{i}}^{\boldsymbol{2}}}\boldsymbol{-}\frac{\boldsymbol{t-}\boldsymbol{t}_{\boldsymbol{m}}}{\boldsymbol{\tau}_{\boldsymbol{i}}}$ | Eq. 3. |
| $\boldsymbol{I}_{\boldsymbol{i}}\boldsymbol{=}\boldsymbol{\Phi}\boldsymbol{(}\boldsymbol{z}_{\boldsymbol{i}}\boldsymbol{)}$ | Eq. 4. |
| $\boldsymbol{z}_{\boldsymbol{i}}\boldsymbol{=}\frac{\boldsymbol{t-}\boldsymbol{t}_{\boldsymbol{m}}}{\boldsymbol{\sigma}}\boldsymbol{-}\frac{\boldsymbol{\sigma}}{\boldsymbol{\tau}_{\boldsymbol{i}}}$ | Eq. 5. |

Where *t* is time, *A* is area, *b* is the parameter determining the mixing of the second EMG (exponentially modified Gaussian) function, *σ* and *t_m_* are the standard deviation and expected value of the common Gaussian component of the two EMG’s respectively, *τ_i_* are the rate parameter of the ith exponential distribution and *Φ* is the cumulative density function of the normal distribution. The initial parameters and their bounds were determined on chromatograms of purified OMV samples. The first peak was modeled with an EMG function and tight constraints were used on the shape and location parameters (*τ_1_, σ, t_m_*). The second peak was modeled with a GEMG function. The lower constrain for the ratio of *τ_i_* /*σ* was set to 0.2 to avoid numerical errors. The Levenberg-Marquardt algorithm in the SciPy python package was used to find the optimal parameters of the two-peak model. All optimizations were repeated 5 times with random initial parameters to avoid local minima. All results were visually evaluated for incorrect fitting.

# Supplementary Tables

| Table S1. Sequences | |
| --- | --- |
| **Name** | **Sequence (5’-3’)** |
| nlpI_fw | GTT AAG GTG ATG GCA ATC AAA AAA GAT TAC |
| nlpI_rv | CAA CCG GGA ACA GGA CGT T |
| lpxM_fw | CAA ACT TGA ACT TAT CAT CAG GCG AAG G |
| lpxM_rv | CGC TAC ACT ATC ACC AGA TTG ATT TTT GC |
| SpC_AIDA_fw | AAA AAA GTC GAC GCC ATG GTT GAT ACC TTA TCA GGT TTA TCA AGT GAG CAA G |
| SpC_AIDA_rv | AAA AAA TCT AGA AAT ATG AGC GTC ACC TTT AGT TGC TTT GCC ATT TAC AGT AAC |
| SpC_Hbp_fw | AAA AAA GAG CTC CGC CAT GGT TGA TAC CTT ATC AG |
| SpC_Hbp_rv | AAA AAA GGA TCC AAT ATG AGC GTC ACC TTT AGT TGC TTT G |
| HSpC_fw | AAA AAA ACA TGT CTA ACA GAA TTT ATT CTC TTC GCT ACA |
| HSpC_rv | AAA AAA GCT AGC TCA GAA TGA ATA ACG AAT ATT AGC G |
| ASpC_fw | AAA AAA ACA TGT CTA ATA AGG CCT ACA GTA TCA TTT GGA |
| ASpC_rv | AAA AAA GAG CTC TCA GAA GCT GTA TTT TAT CCC CAG T |
| SpyCatcher | GTT GAT ACC TTA TCA GGT TTA TCA AGT GAG CAA GGT CAG TCC GGT GAT ATG ACA ATT GAA GAA GAT AGT GCT ACC CAT ATT AAA TTC TCA AAA CGT GAT GAG GAC GGC AAA GAG TTA GCT GGT GCA ACT ATG GAG TTG CGT GAT TCA TCT GGT AAA ACT ATT AGT ACA TGG ATT TCA GAT GGA CAA GTG AAA GAT TTC TAC CTG TAT CCA GGA AAA TAT ACA TTT GTC GAA ACC GCA GCA CCA GAC GGT TAT GAG GTA GCA ACT GCT ATT ACC TTT ACA GTT AAT GAG CAA GGT CAG GTT ACT GTA AAT GGC AAA GCA ACT AAA GGT GAC GCT CAT ATT |

| Table S2. Standardized Uptake Values (SUV) | | | | | |
| --- | --- | --- | --- | --- | --- |
|  | | **SUV (g/ml)** | | | |
|  |  | **SpT-3-NODAGA** | **SpT-23-NODAGA** | **SpT-3-OMV** | **SpT-23-OMV** |
| **3h** | **kidneys** | 2.95 | 2.48 | 1.23 | 1.18 |
|  | **liver** | 0.94 | 0.53 | 6.10 | 5.93 |
|  | **heart** | 0.10 | 0.08 | 0.34 | 0.34 |
|  | **brain** | 0.04 | 0.03 | 0.08 | 0.08 |
|  | **lung** | 0.13 | 0.08 | 0.47 | 0.57 |
|  | **spleen** | 0.10 | 0.14 | 6.89 | 6.01 |
|  | **bladder** | 3.02 | 6.10 | 2.06 | 3.05 |
|  | **intestines** | 0.32 | 0.40 | 0.54 | 0.49 |
| **6h** | **kidneys** | 2.42 | 2.18 | 1.15 | 1.23 |
|  | **liver** | 1.02 | 0.36 | 5.02 | 4.92 |
|  | **heart** | 0.12 | 0.08 | 0.42 | 0.38 |
|  | **brain** | 0.05 | 0.03 | 0.08 | 0.09 |
|  | **lung** | 0.13 | 0.09 | 0.68 | 0.63 |
|  | **spleen** | 0.07 | 0.10 | 5.89 | 5.53 |
|  | **bladder** | 1.60 | 1.03 | 0.99 | 0.87 |
|  | **intestines** | 0.30 | 0.37 | 0.72 | 0.51 |
| **12h** | **kidneys** | 1.93 | 1.75 | 1.32 | 1.29 |
|  | **liver** | 1.01 | 0.35 | 5.46 | 5.01 |
|  | **heart** | 0.11 | 0.09 | 0.30 | 0.31 |
|  | **brain** | 0.04 | 0.03 | 0.09 | 0.09 |
|  | **lung** | 0.14 | 0.08 | 0.56 | 0.53 |
|  | **spleen** | 0.09 | 0.11 | 5.65 | 5.00 |
|  | **bladder** | 0.22 | 0.25 | 0.76 | 0.69 |
|  | **intestines** | 0.29 | 0.24 | 0.78 | 0.56 |

| Table S3. Injected dose (ID) percentages. | | | | | |
| --- | --- | --- | --- | --- | --- |
|  | | **%ID** | | | |
|  |  | **[^64^Cu]SpT-3-NODAGA** | **[^64^Cu]SpT-23-NODAGA** | **[^64^Cu]SpT-3-OMV** | **[^64^Cu]SpT-23-OMV** |
| **3h** | **kidneys** | 14.13% | 9.65% | 2.11% | 1.83% |
|  | **liver** | 5.22% | 2.38% | 49.39% | 47.46% |
|  | **heart** | 0.12% | 0.10% | 0.36% | 0.36% |
|  | **brain** | 0.07% | 0.06% | 0.17% | 0.17% |
|  | **lung** | 0.40% | 0.33% | 1.57% | 1.85% |
|  | **spleen** | 0.06% | 0.05% | 7.37% | 6.55% |
|  | **bladder** | 2.06% | 4.24% | 2.12% | 2.10% |
|  | **intestines** | 4.54% | 3.27% | 4.91% | 4.36% |
| **6h** | **kidneys** | 11.66% | 8.35% | 2.08% | 1.67% |
|  | **liver** | 6.10% | 2.33% | 46.82% | 47.67% |
|  | **heart** | 0.15% | 0.13% | 0.44% | 0.42% |
|  | **brain** | 0.09% | 0.08% | 0.16% | 0.17% |
|  | **lung** | 0.40% | 0.30% | 1.17% | 1.20% |
|  | **spleen** | 0.03% | 0.05% | 6.32% | 6.38% |
|  | **bladder** | 1.25% | 0.54% | 0.64% | 0.39% |
|  | **intestines** | 2.53% | 3.50% | 4.50% | 4.31% |
| **12h** | **kidneys** | 8.21% | 6.38% | 1.61% | 1.87% |
|  | **liver** | 5.84% | 1.82% | 43.45% | 45.58% |
|  | **heart** | 0.12% | 0.12% | 0.29% | 0.36% |
|  | **brain** | 0.08% | 0.09% | 0.19% | 0.17% |
|  | **lung** | 0.49% | 0.27% | 1.58% | 1.26% |
|  | **spleen** | 0.04% | 0.03% | 5.46% | 5.16% |
|  | **bladder** | 0.02% | 0.02% | 0.47% | 0.14% |
|  | **intestines** | 2.99% | 3.00% | 6.37% | 4.20% |

# Supplementary Figures

**Figure S1.**


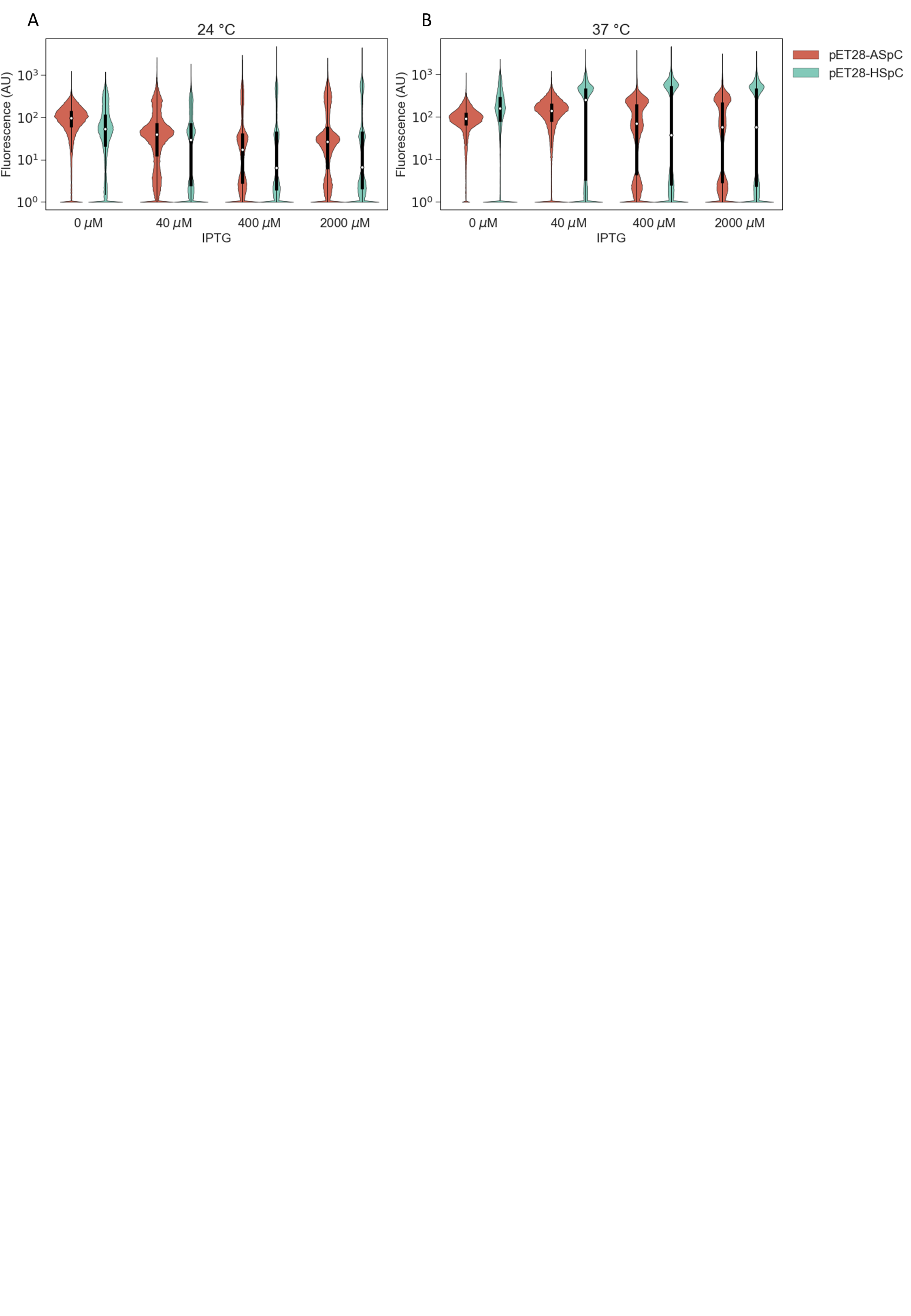


**Figure S1.** Fluorescent intensity distributions of BL21.V cells harboring either pET28-ASpC or pET28-HSpC measured with FCM after induction at different conditions. The white dot of the central boxplot represents the median. Violin plots are scaled to the same width.

**Figure S2.**


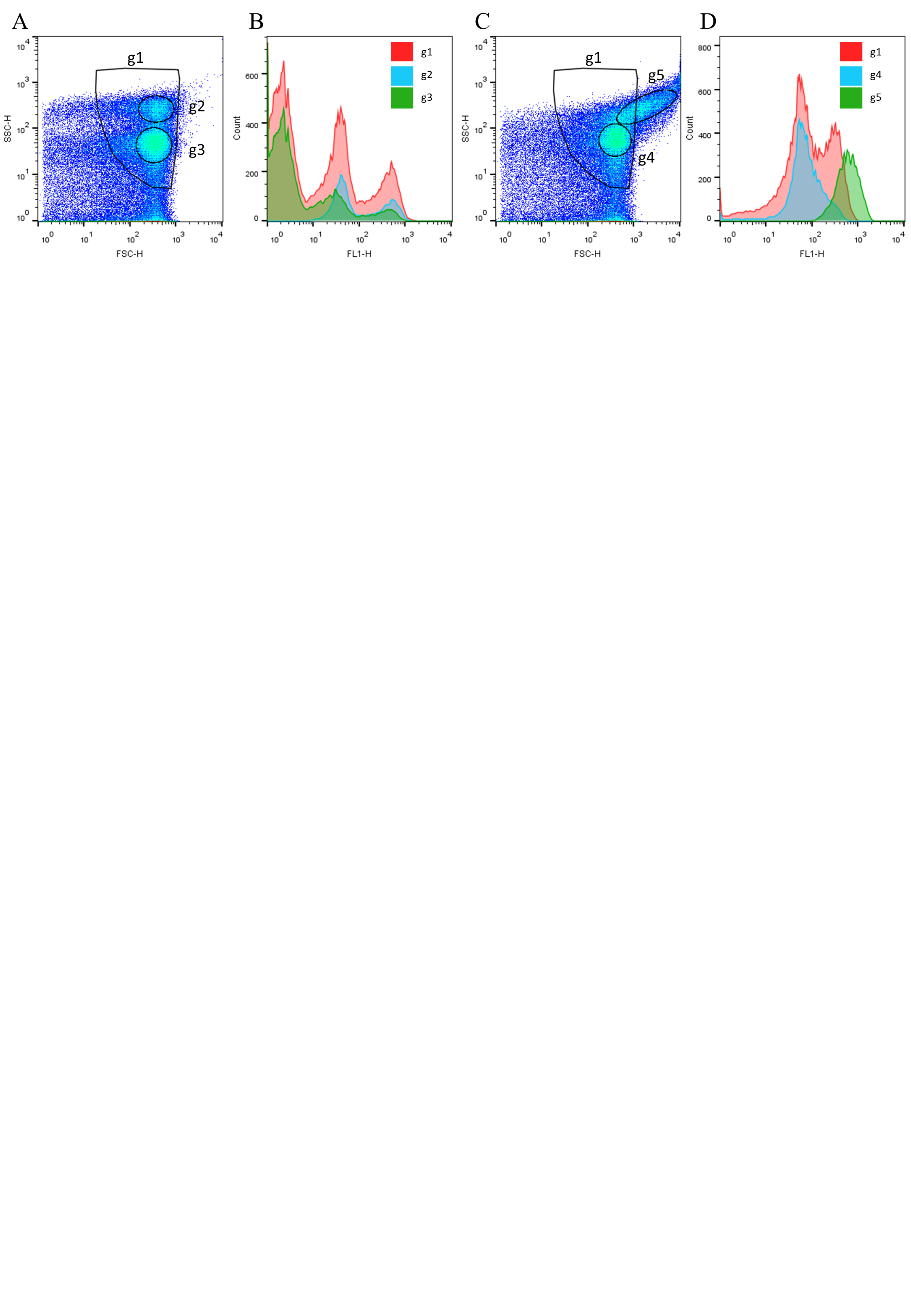


**Figure S2. A)** Flow cytometry FSC/SSC scatter plot of BL21.V harboring pET28-HSpC induced with 400 µM IPTG at 24 °C showing two distinct populations. **B)** Fluorescence intensity histograms corresponding to the gates defined on the same sample show different intensity distributions for the two populations.

**Figure S3.**


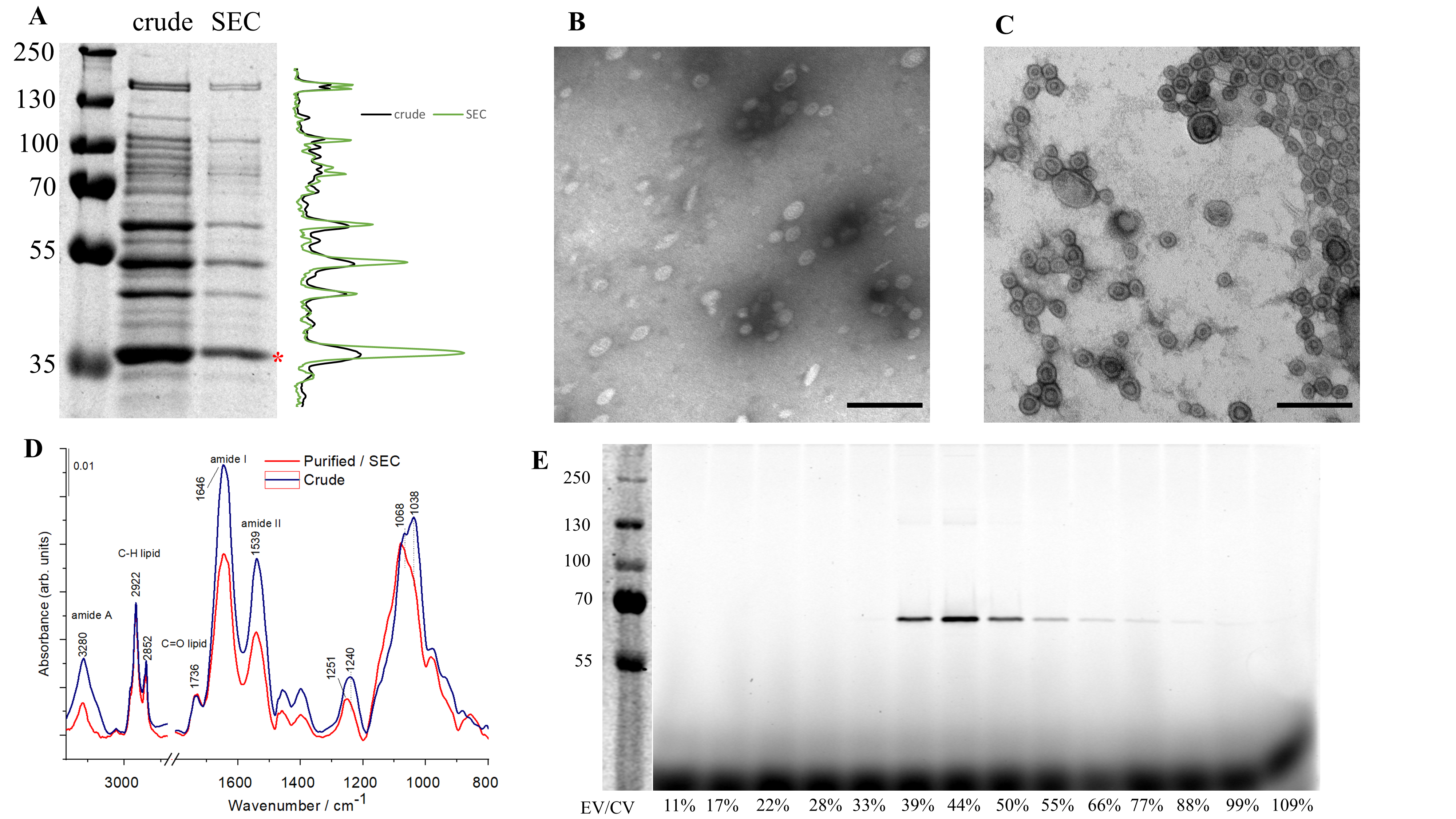


**Figure S3.** Evaluation of Sepharose CL-4B size exclusion chromatography (SEC) for OMV purification. **A)** SDS-PAGE of crude and SEC-purified BL21.V OMVs stained with PageBlue. The density profile of both lanes were normalized to total density and plotted next to the gel photo (black: crude, green: SEC-purified). A large difference corresponding to the OmpF band (red asterisk) is observable suggesting that SEC increases the purity of the OMV sample. An uncropped version of the gel image is presented in Fig. S11. **B)** A TEM photomicrograph of a crude OMV sample showing high levels of background staining. **C)** A TEM photomicrograph of the same isolate after SEC purification. The decrease in background staining is likely due to decreased protein contamination in the sample. **D)** IR spectrum of a BL21.V OMV sample before (blue) and after (red) SEC purification. The relative decrease of amide bands compared to lipid bands indicate that protein contamination is removed during SEC. Spectra are normalized to lipid vibrational bands. **E)** OMVs isolated from BL21.V harboring pET28-ASpC were purified with a CL-4B gravity column. The fractions were incubated with SpT-CF and analyzed using SDS-PAGE. Each lane corresponds to a different fraction, expressed as the percentage of elution volume to column volume (EV/CV). Although most OMVs eluate at ~39-55% CV a small amount is retained and only slowly eluted with the total column volume. The MW marker lane is visualized on a separate channel of the same scan.

**Figure S4.**


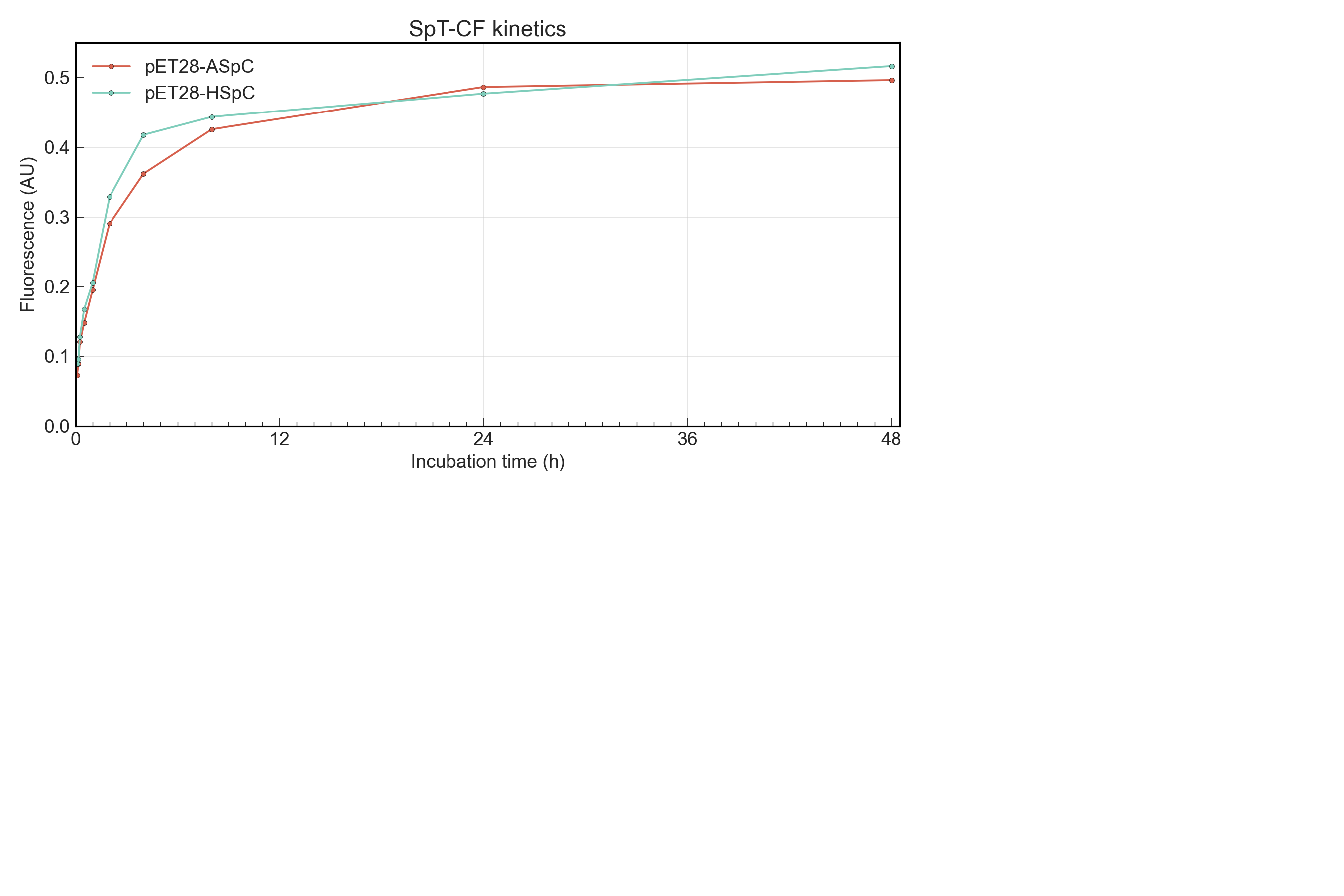


**Figure S4.** SpT-CF binding to the OMV isolates was evaluated at multiple time points using SDS-PAGE. Fluorescence is normalized to OmpF loading control determined after PageBlue staining.

**Figure S5.**


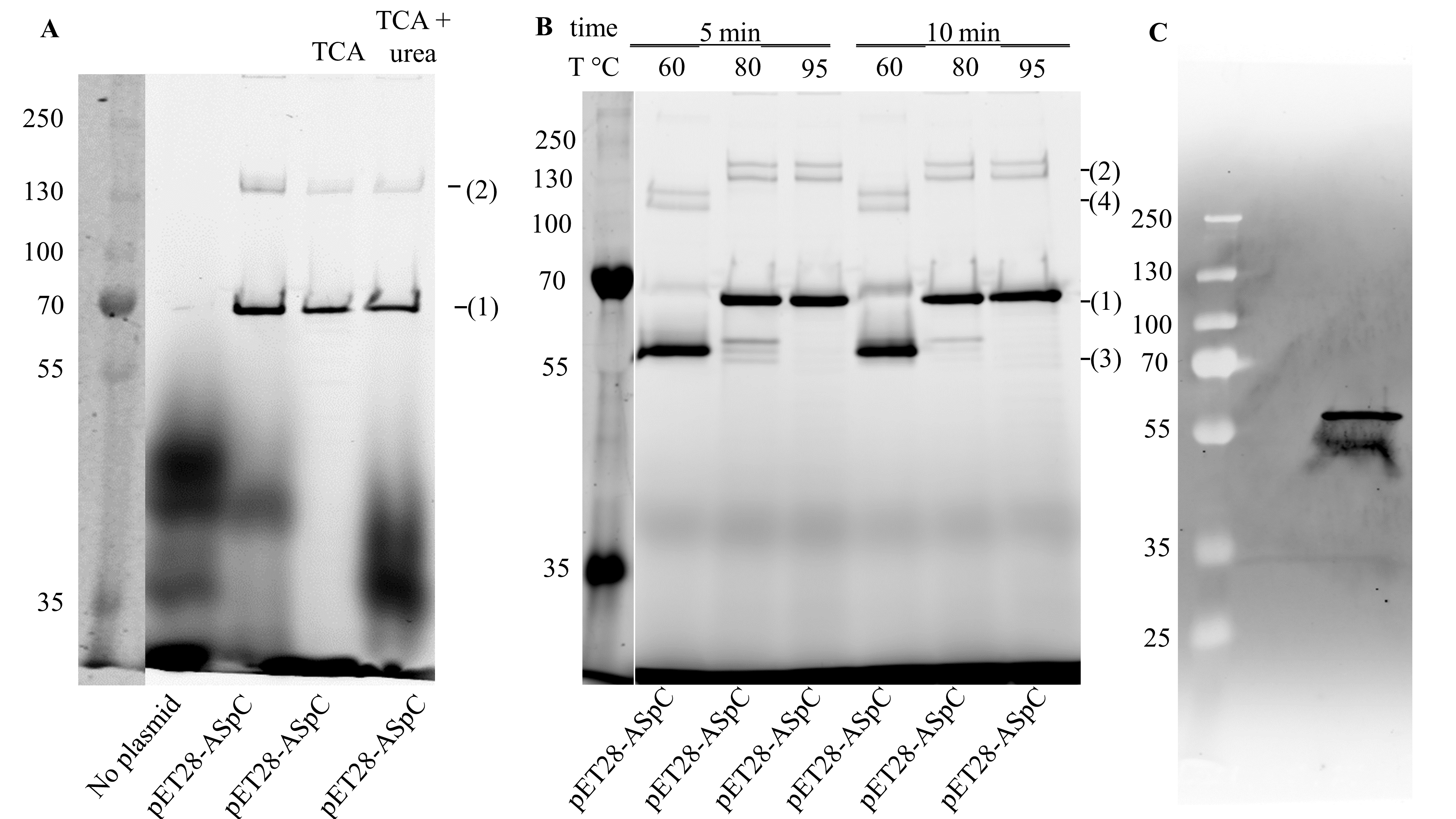


**Figure S5.** OMVs of BL21.V with or without pET28-ASpC were incubated with SpT-CF overnight. **A)** Fluorescent gel image. During sample preparation, 10% trichloroacetic acid (TCA) or 10% TCA + 8 M urea was added to the mixture before heating to 95°C for 5 minutes and loading onto a 10% polyacrylamide gel. OMVs isolated from a plasmidless culture show no SpT-CF binding. An unidentified band (2) is observable with approximately twice the MW of AIDA-SpC (1). **B)** Fluorescent gel image. The unidentified band (2) displays similar heat modification properties to AIDA-SpC (1) when the duration and temperature of the heating step during sample preparation is varied resulting in a shift towards an apparently smaller MW (3 and 4). **C)** OMVs were isolated from BL21.V harboring pAIDA1. The unidentified band is not observable on a Western blot labeled with a fluorescent anti-His antibody. The MW markers of A) and B) are visualized on a separate channel of the same scan.

**Figure S6.**

**
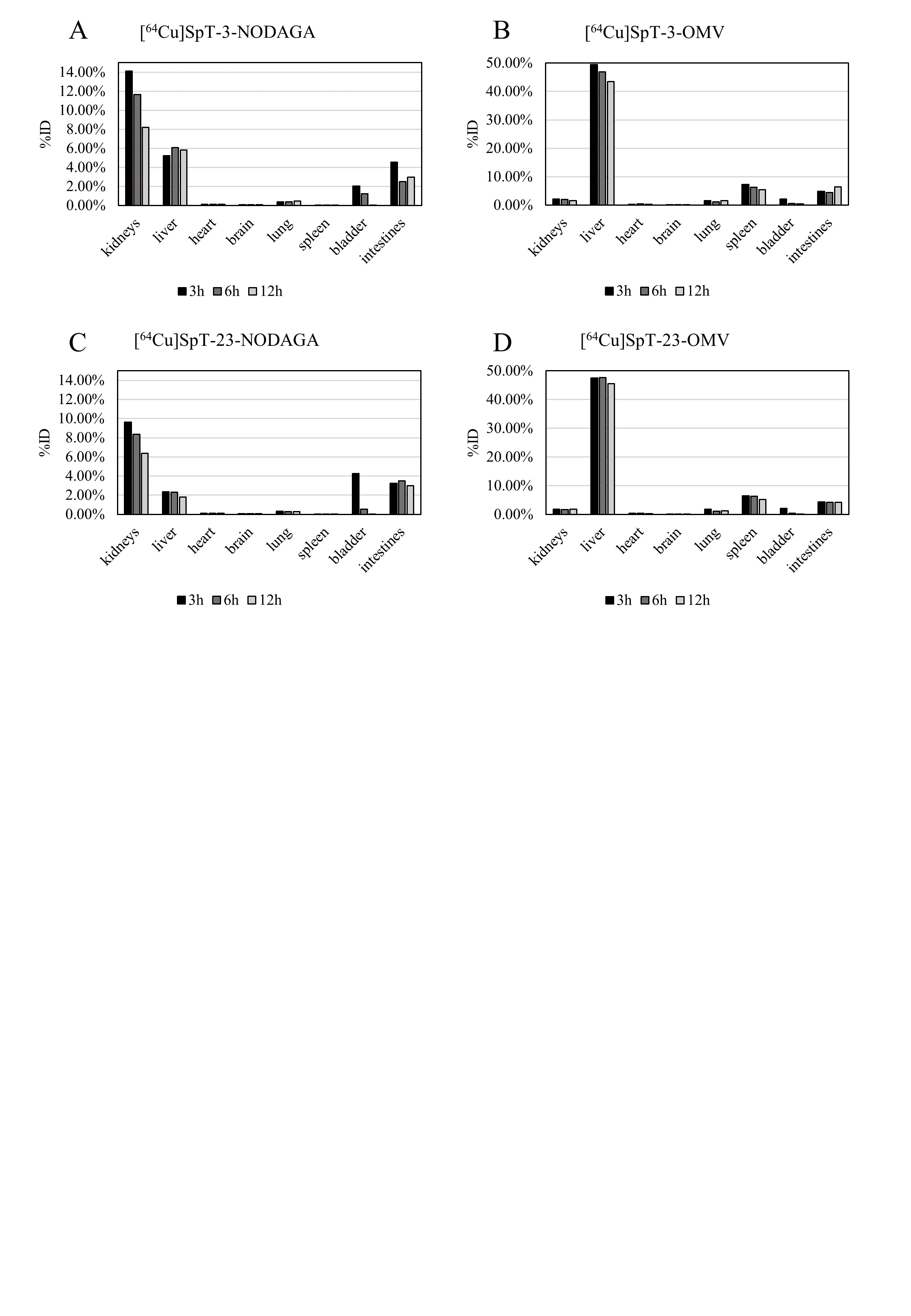
**

**Figure S6.** Organ uptakes determined with PET/MRI expressed as percentage of injected dose. **A)** Biodistribution of SpT-3-NODAGA labeled with ^64^Cu 3, 6, and 12 hours post injection. **B)** Biodistribution of OMVs labeled using SpT-3-NODAGA 3, 6, and 12 hours post injection. **C)** Biodistribution of SpT-23-NODAGA labeled with ^64^Cu 3, 6, and 12 hours post injection. **D)** Biodistribution of OMVs labeled using SpT-23-NODAGA 3, 6, and 12 hours post injection.

**Figure S7.**

**
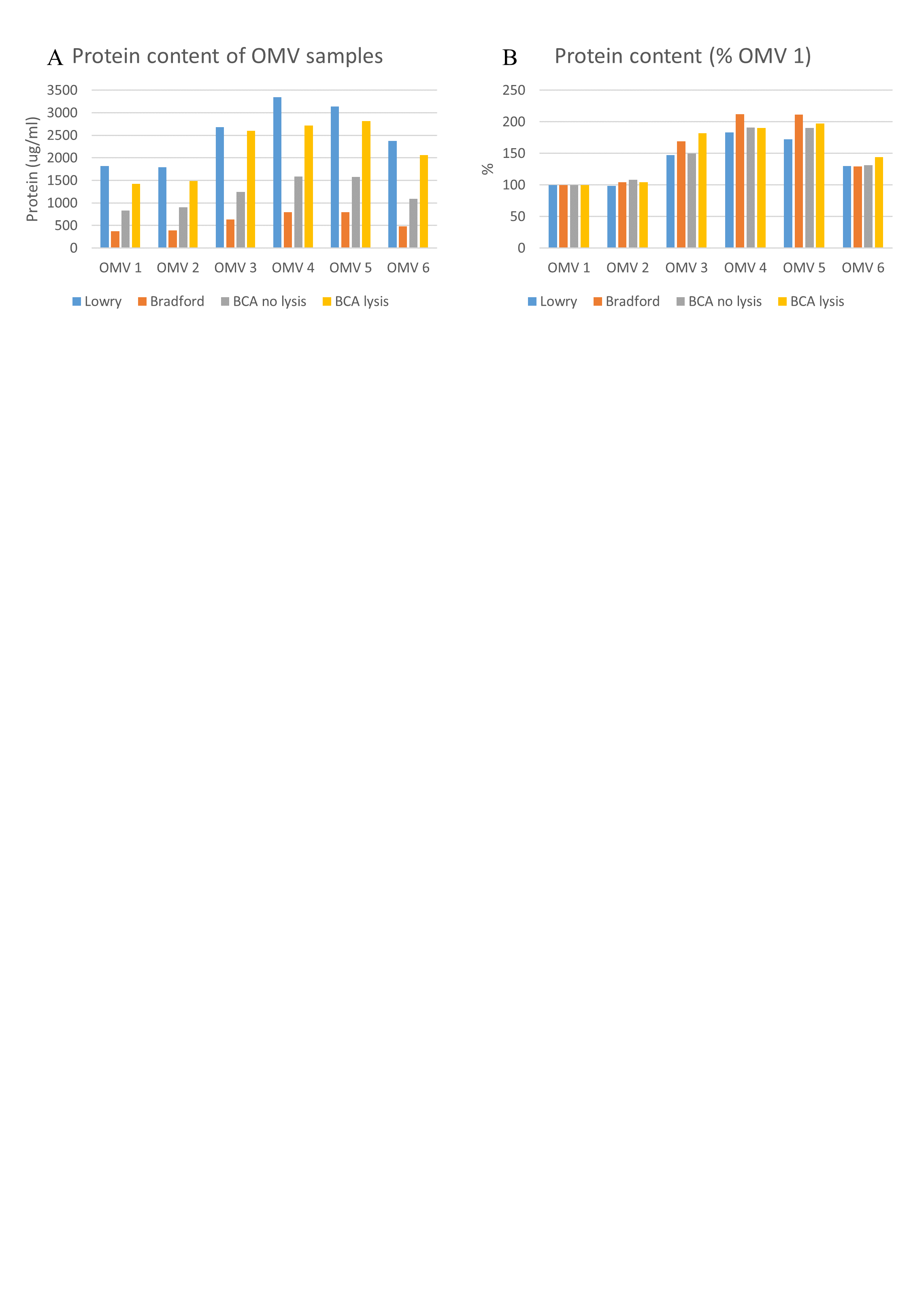
**

**Figure S7.** Protein content of six SEC-purified OMV samples measured with four different methods. Lowry: Pierce Modified Lowry Protein Assay kit (Thermo Scientific), Bradford: Quick Start Bradford 1x Dye Reagent (Bio-Rad), BCA: Pierce BCA Protein Assay Kit - Thermo Fisher. BCA assay was carried out on intact and lysed OMVs, where lysis was facilitated by the addition of 1% Triton X-100 and 0.1% SDS to the samples and incubating for 30 minutes on ice. The same BSA standard was used for Lowry, Bradford and BCA without lysis. A separate BSA dilution series was prepared for BCA with lysis by the addition of 1% Triton X-100 and 0.1% SDS. **A)** Protein concentration of different OMV samples. **B)** Protein concentration relative to the first sample.

**Figure S8.**

**
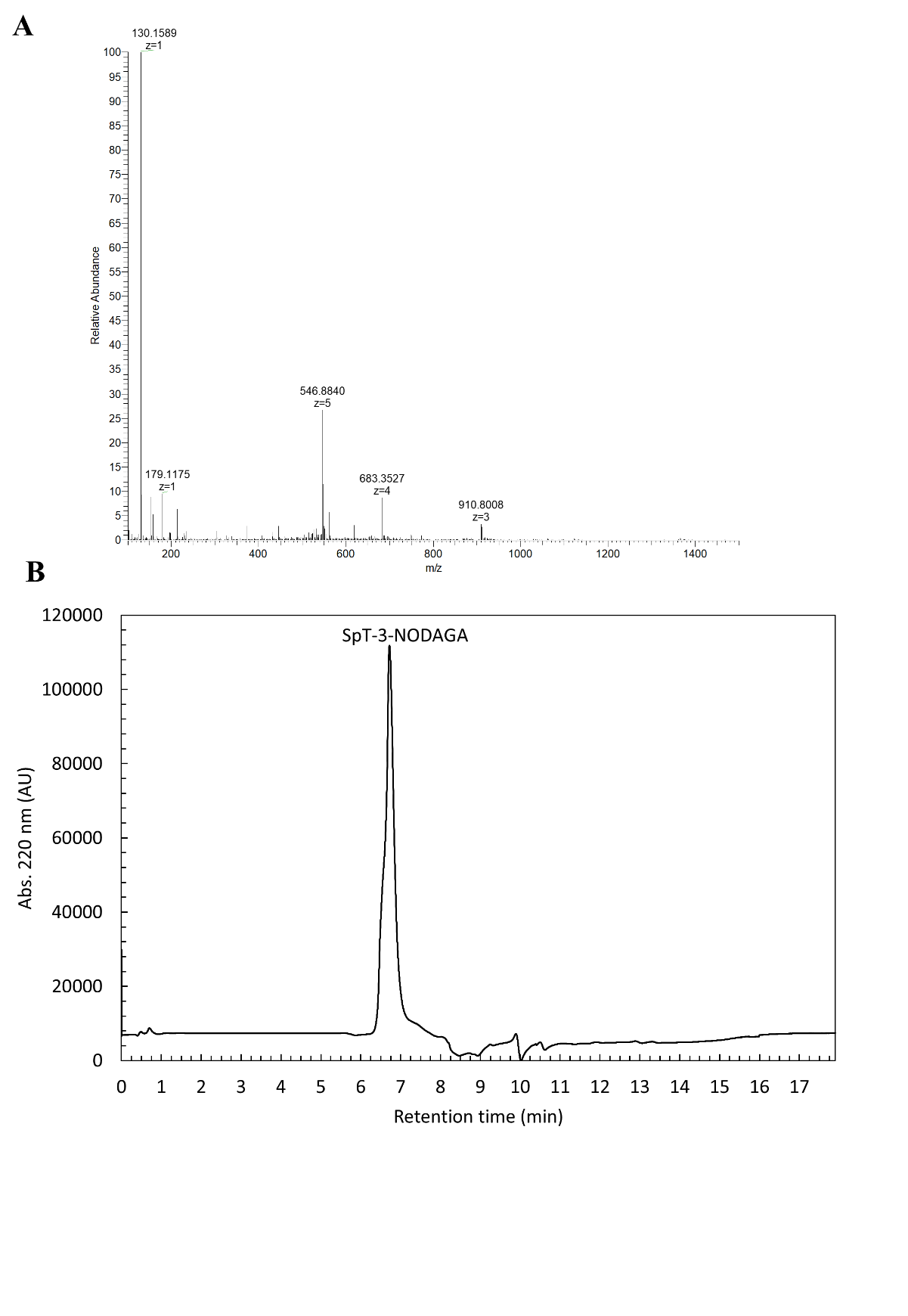
**

**Figure S8. A)** Mass spectrum of SpT-3-NODAGA. m/z: [M+3H]3+: 911.0153 (calc: 911.0845), [M+4H]4+: 683.5026 (calc: 683.5652), [M+5H]5+: 546.9894 (calc: 547.0536); and the exact monoisotopic mass is (calc/meas): 2730.2318Da/2730.05080Da. **B)** RP-HPLC chromatogram of SpT-3-NODAGA (tR = 6.720 min).

**Figure S9.**

**
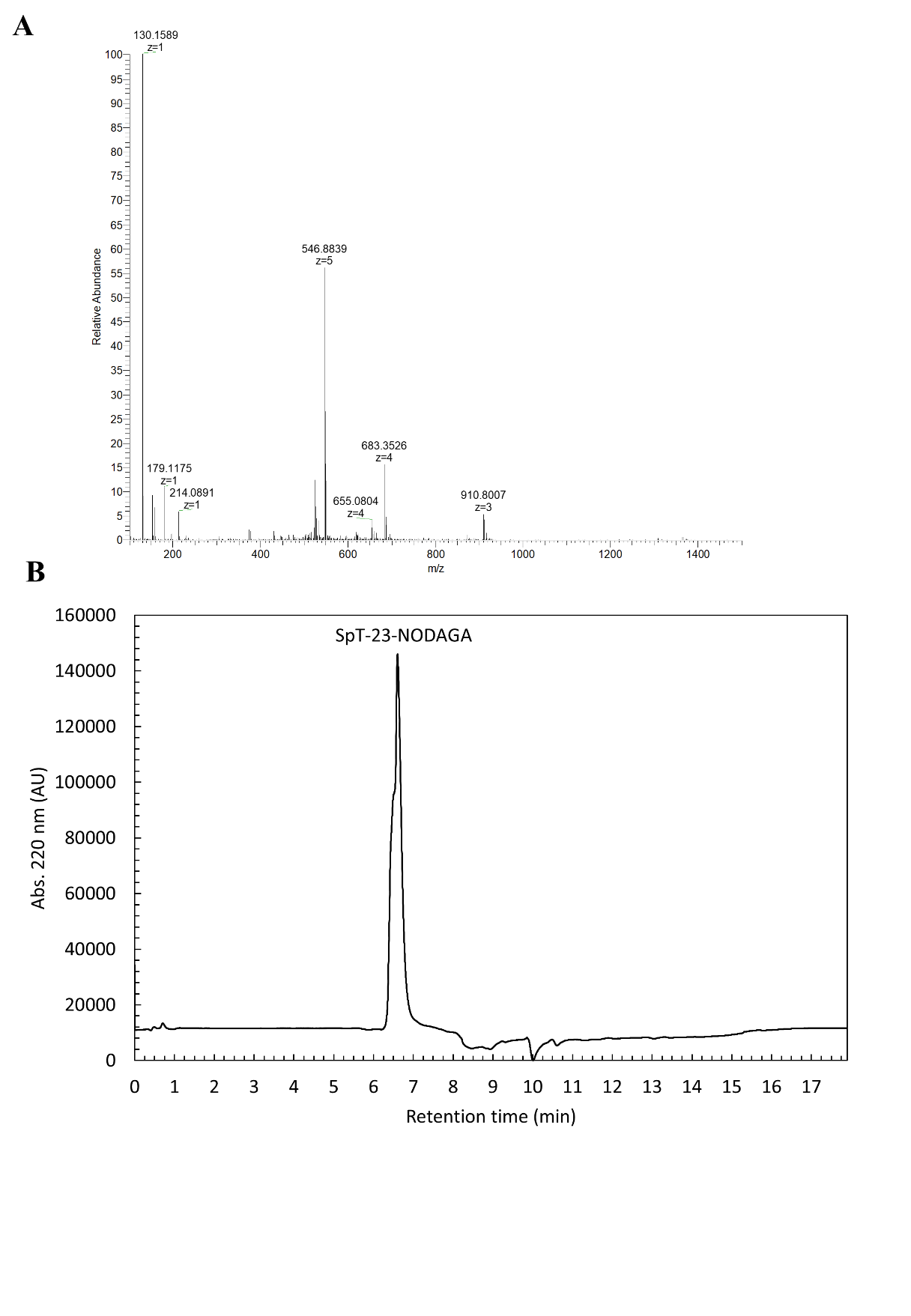
**

**Figure S9. A**) Mass spectrum of SpT-23-NODAGA. m/z: [M+3H]3+: 911.0375 (calc: 911.0845), [M+4H]4+: 683.5127 (calc: 683.5652), [M+5H]5+: 546.9960 (calc: 547.0536); and the exact monoisotopic mass is (calc/meas): 2730.2318Da/2729.4030Da. **B)** RP-HPLC chromatogram of SpT-23-NODAGA (tR = 6.603 min).

**Figure S10.**

**Figure S10. A)** Mass spectrum of SpT-CF. m/z: [M+2H]2+: 1094.5146 (calc: 1094.4764), [M+3H]3+: 730.0122 (calc: 729.9884), [M+4H]4+: 547.7615 (calc: 547.7356); and the exact monoisotopic mass is (calc/meas): 2186.9910Da/2187.0148Da. **B)** RP-HPLC chromatogram of SpT-CF (tR = 7.767 min).

**Figure S11.**


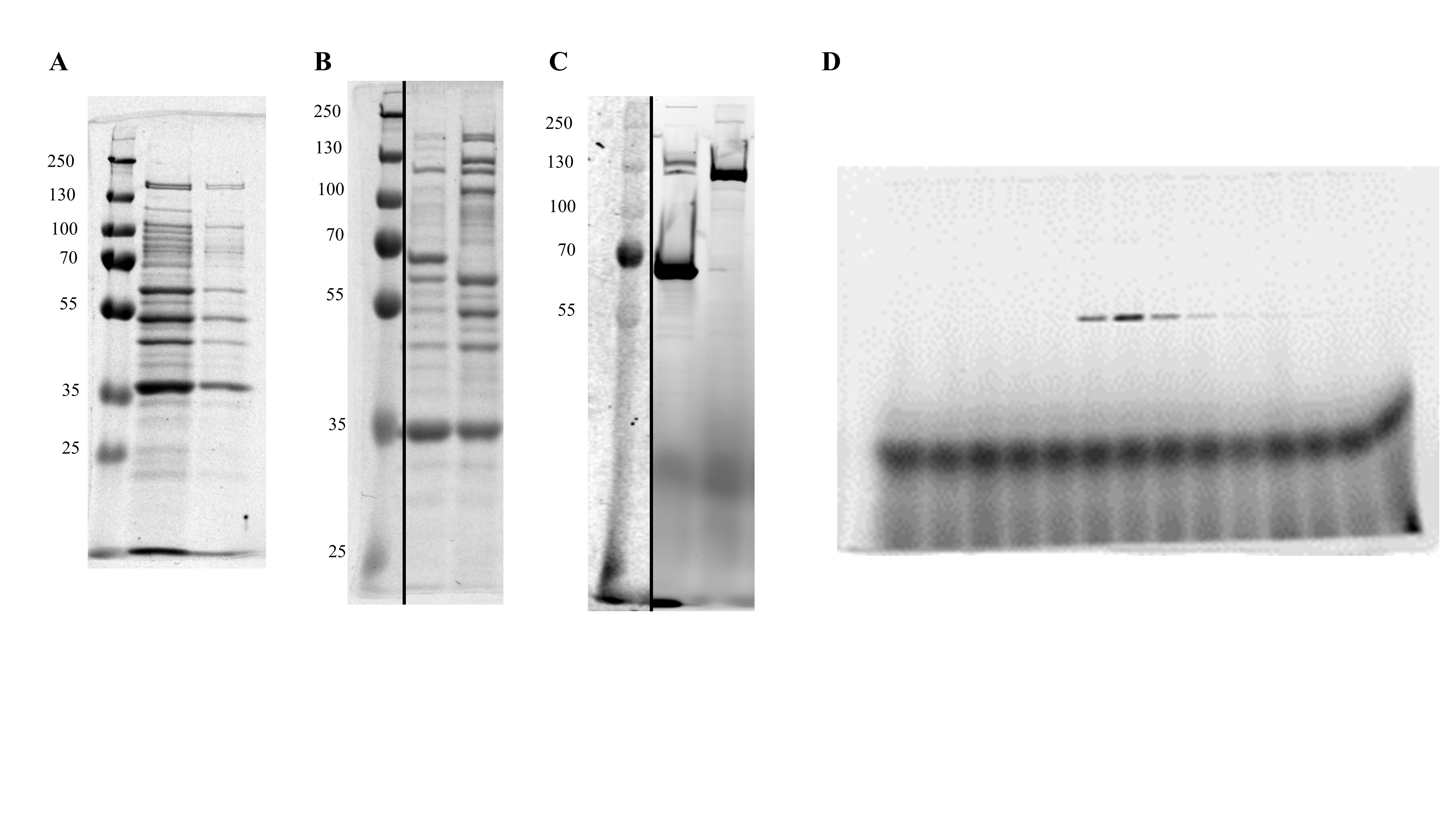


**Figure S11.** Full length gel images. **A)** Full length image corresponding to Fig 1. D and Fig. S3. A. **B) and C)** Full length images corresponding to the left and right halves of Fig. 2. B, respectively. Some lanes were cut between the marker and the lanes of interest. This is indicated by a vertical black line. **D)** Full length image corresponding to Fig. S3.E (fluorescein channel).

# References

1. Zhang Z, Kuipers G, Niemiec L, Baumgarten T, Slotboom DJ, de Gier JW, et al. High-level production of membrane proteins in E. coli BL21(DE3) by omitting the inducer IPTG. Microb Cell Fact. 2015; 14: 142.

2. Kochan K, Perez-Guaita D, Pissang J, Jiang JH, Peleg AY, McNaughton D, et al. In vivo atomic force microscopy-infrared spectroscopy of bacteria. J R Soc Interface. 2018; 15.

3. Jensen SI, Lennen RM, Herrgard MJ, Nielsen AT. Seven gene deletions in seven days: Fast generation of Escherichia coli strains tolerant to acetate and osmotic stress. Sci Rep. 2015; 5: 17874.

4. Jensen SI, Nielsen AT. Multiplex Genome Editing in Escherichia coli. Methods Mol Biol. 2018; 1671: 119-29.
